# Structural Investigation of MscL Gating Using Experimental Data and Coarse Grained MD Simulations

**DOI:** 10.1371/journal.pcbi.1002683

**Published:** 2012-09-20

**Authors:** Evelyne Deplazes, Martti Louhivuori, Dylan Jayatilaka, Siewert J. Marrink, Ben Corry

**Affiliations:** 1 School of Biomedical, Biomolecular and Chemical Sciences, The University of Western Australia, Perth, Australia; 2 Groningen Biomolecular Sciences and Biotechnology Institute, and Zernike Institute for Advanced Materials, University of Groningen, Groningen, The Netherlands; 3 Research School of Biology, The Australian National University, Canberra, Australia; Stockholm University, Sweden

## Abstract

The mechanosensitive channel of large conductance (MscL) has become a model system in which to understand mechanosensation, a process involved in osmoregulation and many other physiological functions. While a high resolution closed state structure is available, details of the open structure and the gating mechanism remain unknown. In this study we combine coarse grained simulations with restraints from EPR and FRET experiments to study the structural changes involved in gating with much greater level of conformational sampling than has previously been possible. We generated a set of plausible open pore structures that agree well with existing open pore structures and gating models. Most interestingly, we found that membrane thinning induces a kink in the upper part of TM1 that causes an outward motion of the periplasmic loop away from the pore centre. This previously unobserved structural change might present a new mechanism of tension sensing and might be related to a functional role in osmoregulation.

## Author Summary

Cells in biological organisms have to be able to respond to mechanical forces during processes such as touch, hearing, pain sensation and tissue growth. One way this is achieved is through mechanosensitive ion channels, membrane embedded proteins that initiate electrical signalling upon tension within the cell or cell membrane. The malfunction of such channels is also associated with a range of diseases including muscular dystrophy and cardiac arrhythmia. In this manuscript, we study in detail the mechanosensitive channel of large conductance (MscL) from bacteria, a model system in which to understand the principles of mechanosensation. Despite many years of investigative work the details of how the protein senses tension in the surrounding membrane remain unknown. By combining structural data from experiments with computer simulation we are able to model the open channel structure of the protein and report previously unobserved structural changes that might present a new mechanism of sensing tension. The methods developed in this paper are not limited to the study of mechanosensitive ion channels and may be useful in understanding the structure and function of other membrane proteins.

## Introduction

Mechanosensitive ion channels are ubiquitous membrane proteins that enable a cell to respond to deformation forces in the surrounding lipid bilayers or cytoskeleton. This process, known as mechanosensation, is thought to have evolved to protect bacterial cells from sudden osmotic shock [1], [2]. In eukaryotes, mechanosensation is involved in a physiological processes including hearing, touch sensation and gravitropism [2]. Shortly after the discovery of mechanosensitive ion channels in *Escherichia coli* bacteria [3] the gene of the mechanosensitive ion channel of large conductance (Eco-MscL) was identified and cloned [4]. The crystal structure of the closed pore MscL [5] from *Mycobacterium tuberculosis* (Tb-MscL) revealed a homo-pentameric channel where each subunit consists of two transmembrane (TM) helices, TM1 and TM2, connected by an extracellular loop and cytoplasmic N- and C-terminal. In the closed state, the TM1 helices are tightly packed to form a narrow constriction referred to as the hydrophobic gate. Gating is induced by tension in the surrounding lipid bilayer that triggers a large conformational change to form an open channel of approximately 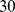 Å diameter [6]–[9]. The structure and function of MscL, have been investigated extensively using a range of techniques including patch clamp studies (see [10], [11] and [2] for reviews), mutation studies [12]–[16], FRET [9], [17], EPR spectroscopy [7], [18], [19], structural modelling [20], [21] and MD simulations [22]–[32]. Based on the original crystal structure [5] and the large conductance of the pore it was initially thought the open pore is lined by all 10 TM helices. A series of EPR experiments [7], [18], [19] resulted in a revised model of the open pore that is mostly lined by TM1. This can only be achieved by a large tilting motion of the TM helices which allows them to cover the increased surface of the pore. The suggested gating mechanism involves an iris-like opening that creates an open pore with a significant decrease in membrane span. A large number of experimental and computational studies have provided insight into the structure and function of MscL but there remain questions about the details of the open pore structure and the gating mechanism. It is also unclear how the channel senses the tension in the membrane that subsequently triggers gating. Answering these questions is essential for our understanding of mechanosensation at the molecular level in both bacterial and eukaryotic cells, and to aid in the design of engineered channels with novel functionalities [33].

Molecular Dynamics (MD) simulations are well suited to examine the structure and dynamics of proteins but atomistic MD simulations of membrane proteins are computationally expensive and are commonly restricted to 10's or 100's of ns. This timescale is considerably shorter than most physiological processes such as channel gating, which often take place in the millisecond range. Furthermore, the large conformational changes associated with ion channel function are often separated by significant energy barriers and long simulations are necessary to ensure sufficient conformational sampling. Different approaches have been used to address the timescale and sampling issues of standard atomistic MD simulations. One way is to apply external forces to the protein to induce or accelerate the wanted conformational change. A number of studies used different forms of surface tension or negative pressure on the membrane and/or radial forces applied to the protein to model the gating of MscL [23]–[28]. From these simulation studies it is evident that long simulations are needed to obtain an open pore of the MscL protein without the use of radial forces or large tensions. An alternative way of addressing the sampling issue it to use restraints based on experimental data which directs the evolution of the system through the conformational space as demonstrated by several studies to model the structure of membrane proteins [7], [9], [34], [35]. The timescale issue of MD simulations can be addressed by using coarse graining (CG). The idea is to group several atoms into a single particle which reduces the system size and removes the fastest degrees of freedom such that a larger time step can be used, making simulations in the 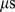 range feasible (see [36], [37] for a review of CG models for proteins). In addition, CG simulations also show some enhanced sampling efficiency as a direct result of the reduced number of effective interactions between particles. Yefimov et al. [29] used CG MD simulations to model the WT and mutants of Tb-MscL with and without tension. Several simulations of multiple 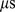 were carried out and produced an expanded and stable water filled pore. The tension used was lower than in previous simulation studies of MscL but still exceeded the tension that is required to open the pore under physiological conditions. Here we combine CG MD simulations with experimental restraints by incorporating inter-subunit distances and solvent accessibility data from EPR and FRET experiments into a CG model of MscL. The aim of this study is to obtain an open pore structure of the MscL protein that is consistent with experimental data and thus gain insight into the structural re-arrangements during gating. Multiple simulations in the 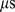 range allowed us to achieve much greater conformational sampling and observe structural changes that were not seen in single shorter simulations. By combining CG simulations with experimental restraints we were able to induce gating and model the open pore structure of MscL without using excessive tension. Furthermore, we observed previously unseen structural changes that may have an important functional role.

## Methods

### Overview of simulation approach

**Figure 1:**
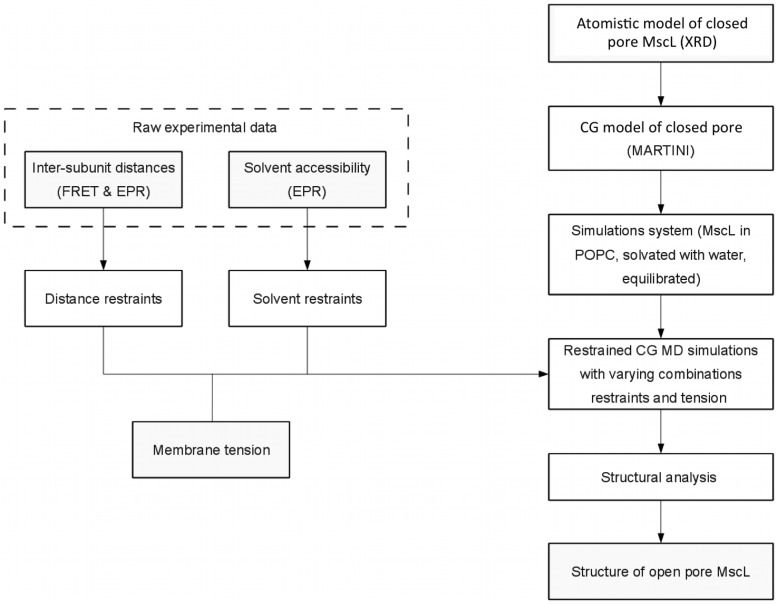
Overview of the simulation approach outlining the individual steps involved to obtain the final set of open pore structures.

Figure 1 provides an overview of the simulation approach. The starting structure of the closed state MscL protein was an atomistic homology model of Eco-MscL [38] which is based on the crystal structure of Tb-MscL [5] and the structural models of Sukharev et al [20], [21]. The simulation system was modelled using MARTINI, a biomolecular force field for CG simulations [39], [40]. In the MARTINI model four heavy atoms are, on average, represented by a single CG particle. All non-bonded particles interact via a Lennard-Jones potential energy function and the strengths of this interaction is used to mimic the chemical nature of the different particle types. In addition, charged particles interact via a Coulombic energy function. The MARTINI model has been successfully used to model lipid membranes [41]–[43] and membrane proteins [29], [30], [32], [44], [45]. Note, secondary structure is maintained in the MARTINI model using standard bonded potentials. An additional elastic network was not used.

An equilibrated CG structure of the closed state with water and lipid was used as the starting structure for all simulations. The inter-subunit distances and the solvent accessibility data from EPR and FRET experiments were converted into restraints and incorporated into the CG model. A series of simulations with different combinations of solvent and distance restraints and tension were carried out. Two different tensions were used: 12 dynes/cm, the tension required to induce gating in patch clamp experiments of MscL [8], [46], and 30 dynes/cm. The latter value was used as experience with previous CG MD simulations of MscL showed that a larger tension was needed to induce gating [29], [30].

The different simulation protocols are summarised in Table 1. Simulations without any restraints (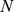) and with tension only (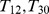) were mainly used as references for comparison during data analysis. Different combinations of using the full set of EPR and FRET distance restraints with and without tension were carried out. In addition, simulations with a reduced set of distance restraints were carried out, in which 10% of randomly selected restraints were removed. For each protocol we aimed to collect a minimum of two trajectories to ensure reproducibility. In all restrained simulations, both distance and solvent restraints were introduced over a period of 1000 ns. After that, the simulation was continued for another 500 ns with the restraints in place. For simulations with distance restraints this was followed by a further 500 ns where the distance restraints were removed.

**Table 1:** Overview of Simulation protocols listing the different combinations of EPR and FRET distance restraints, solvent restraints and tension.

Protocol	Type of restraints	Tension (dynes/cm)	Label
None	None	No	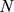
Tension	None	12	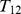
		30	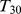
Distance	EPR+FRET	No	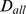
	EPR	No	* 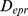
	FRET	No	* 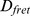
	EPR+FRET, reduced a	No	* 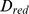
Distance+tension	EPR+FRET	12	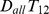
	EPR+FRET	30	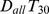
	EPR	12	* 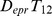
	EPR	30	* 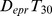
	FRET	12	* 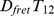
	FRET	30	* 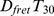
	EPR+FRET, reduced a	12	* 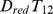
	EPR+FRET, reduced a	30	* 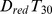
Solvent	all solvent restraints	No	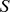
Solvent+tension	all solvent restraints	12	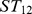
		30	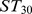
Solvent+distance	EPR+FRET	No	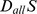
Solvent+distance+tension	EPR+FRET	12	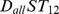
	EPR+FRET	30	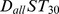
None (DMPC)	None	No	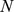 DMPC

If not stated otherwise, simulations were carried out in POPC lipids.

a 10% of randomly selected restraints were removed.

* indicates that only a subset of the distance restraints were used.

Simulations were carried out using GROMACS 4.0.4 [47] with a 25 fs time step. The protein was embedded in a 1-Palmitoyl-2-oleoylphosphatidylcholine (POPC) lipid bilayer and solvated with water. The final simulation system consisted of 1 MscL protein (1435 particles), 1152 POPC lipids (14'276 particles) and 23'868 water particles bringing the total particle count to 40'279. The simulation system was contained in a rectangular box of dimension 20×20×12 nm and periodic boundary conditions were applied in all directions. The system was equilibrated with backbone particles restrained to their starting position for 20 ns followed by an unrestrained simulation of 10 ns. The system was simulated in an NPT ensemble at 310 K using a Berendsen temperature coupling scheme with a time constant of 0.25 ps. Berendsen pressure coupling with a reference pressure of 1 bar and a time constant of 0.5 ps was used. For simulations without tension, a semi-isotropic pressure coupling was used with a reference pressure of 1 bar in the x/y and z direction. Tension was simulated using a surface-tension pressure coupling scheme where the surface tension is coupled to the x/y dimension to simulate constant lateral tension in the membrane while normal pressure coupling is used in the z direction. For each simulation a final average protein structure was calculated by minimising the protein structure obtained from the average position of all backbone particles from the last 50 ns of simulation. The average structures were only used for visual inspection of kinks in TM1 but not for the quantitative analysis, the calculation of RMSD values, pore radius or radial position. Details on analysis protocols are given in the Text S1.

In addition to the simulations in a POPC lipid bilayer we carried out simulations of the MscL protein in short-chain lipids. The simulation system was identical to the one used for restrained simulations in POPC except that the POPC lipids were replaced by Dimyristoyl phosphatidyl choline (DMPC) lipids. POPC lipids contain a 16 carbon and a 18 carbon-chain tail while DMPC has two tails with 14 carbons each. The simulations in DMPC did not contain solvent restraints or distance restraints.

### Implementation of restraints

**Figure 2:**
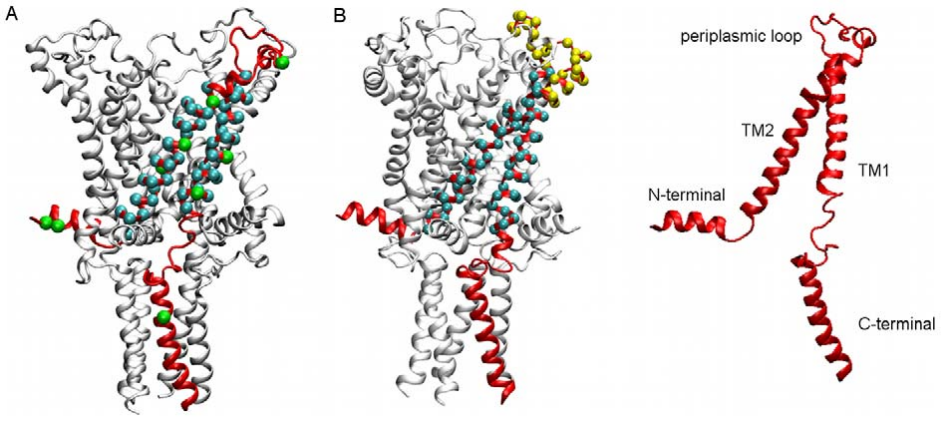
Location of residues of distance and solvent restraints. For clarity, the residues are only shown on 1 of the 5 subunits but restraints were applied to all 5 subunits. A: inter-subunit distances from FRET experiments [9] (green) are located across the protein. Inter-subunit distances from EPR experiments [7] (blue) are located in the transmembrane helices TM1 and TM2. B: Solvent accessibility data from EPR experiments for residues in the transmembrane helices TM1 and TM2 [7] (blue) and the periplasmic loop [38] (yellow).

Table 2 summarises the raw experimental data used for the restraints while Figure 2 depicts the position of all residues for both the solvent and distance restraints. EPR experiments provided data for lipid and water accessibility and inter-subunit distances for residues in the TM region [7] and accessibility data for the periplasmic loop [38]. Additional inter-subunit distances for selected residues across the protein were obtained from FRET spectroscopy [9].

**Table 2:** Overview of experimental data used for distance and solvent restraints in the simulations.

Type of data	Source of data	Location	Restraints
inter-subunit distances	EPR [7]	29 residues in TM1, 36 residues in TM2	650 distance restraints^a^
inter-subunit distances	FRET [9]	9 residues across protein	90 distance restraints a
lipid and water accessibility	EPR [7]	29 residues in TM1, 25 residues in TM2	215 solvent restraints^b^
lipid and water accessibility	EPR [38]	28 residues in periplasmic loop	50 solvent restraints c

Positions of restraints are shown in Figure 2.

a each inter-subunit distance results in 10 distance restraints based on the pentameric structure of MscL.

b only 43 of the 54 residues showed significant changes in combined lipid and water accessibility resulting in 215 solvent restraints.

c only 10 of the 28 residues showed significant changes in combined lipid and water accessibility resulting in 50 solvent restraints.

Distance restraints representing distance between equivalent residues in each subunit from FRET were implemented using a flat bottom harmonic potential where the target distance is equal to the inter-subunit distance in the open state obtained from FRET experiments and the width is equal to the experimental uncertainty. The EPR distance restraints were implemented using harmonic half potentials as all inter-subunit distances in the open state are 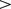
15 Å. Based on the geometry of the pentameric structure of MscL each inter-subunit distance resulted in 10 distance restraints. A total of 90 distance restraints from FRET and 650 distance restraints from EPR were incorporated into the GROMACS topology of the CG model of MscL using tabulated potentials.

Solvent restraints were based on the lipid and water accessibility measurements of residues in the TM helices and the periplasmic loop from EPR experiments. The raw experimental data was converted into restraints by changing the interactions of the side chain particles with water to represent the change in solvent exposure of selected residues upon opening of the channel. Although lipid and water accessibility are separate measurements in EPR experiments, they are conceptually related. To simplify the implementation, the data from lipid and water accessibility were combined into a single parameter 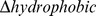 which represents a change in hydrophobic character of a given residue during gating of the channel. Using the reported results from 54 residues in TM1 and TM2 [7] and 28 residues in the periplasmic loop [38] 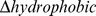 was determined for each of these residues and incorporated into the MARTINI force field [39]. We created a new set of particles that showed stronger or weaker interactions with water particles but unaltered interactions with all other particle types. These new particle types were used to change interactions between the side chain particles of selected residues and water particles based on their 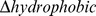 value (see Tables S1 to S3 in Text S1).

The final simulation system contained 740 distance restraints and 265 solvent restraints that represent a change in inter-subunit distances and solvent exposure between the closed and the open channel. It was important to slowly introduce the restraints rather than impose a sudden change onto the closed pore. This was achieved using the coupling parameter 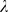 from the free energy perturbation method in GROMACS. Further details of the implementation of the restraints are given in Text S1.

## Results

We carried out a large number of simulations with varying combinations of tension and solvent and distance restraints. The simulations using a reduced set of distance restraints (marked * in Table 1) were used to test the effect of restraints and ensure the structural changes observed were independent of restraints on specific residues. Only simulations using the full set of distance restraints (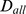, 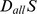, 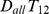, 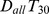, 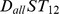, 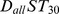) were included in the selection of potential open pores. In general, two trajectories were carried out for each protocol but simulations combining solvent restraints and distance restraints in the absence of tensions (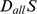) required 4 attempts to obtain a final structure. This suggests that it is hard to find a structure that is consistent with experimental data in the absence of tension, reflecting the physiological condition of the open channel. Simulations combining distance and solvent restraints with the higher tension (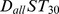) proved to be unstable and were discontinued suggesting that the combination of high tension and full set of restraints exerts too much force on the protein.

### Effect of restraints

**Figure 3:**
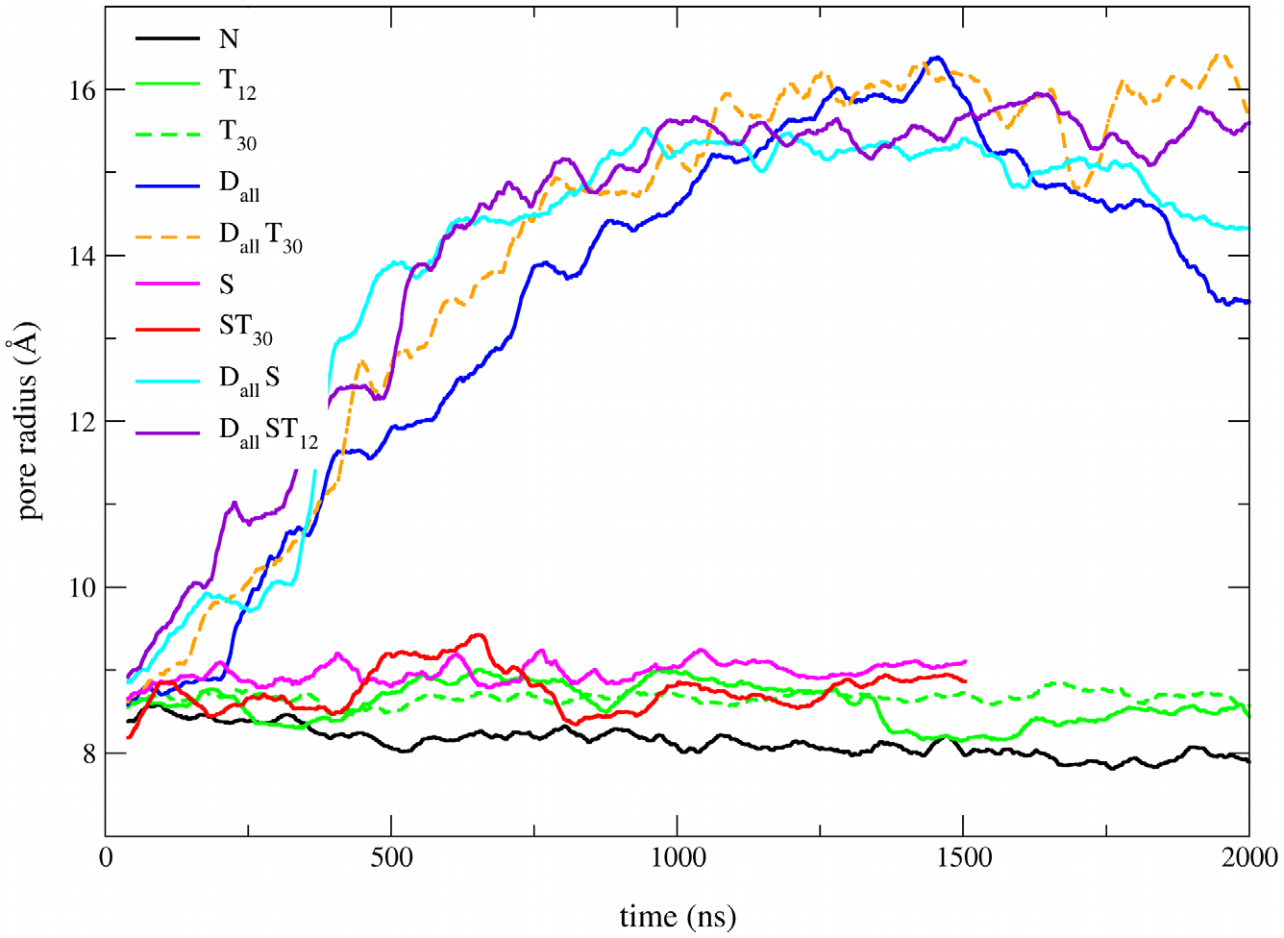
Pore radius as a function of simulation time for some of the different simulation protocols combining distance and solvent restraints with tension. The pore radius at the hydrophobic gate was estimated by the radius of the circle inscribed in the pentagon formed by the 5 subunits, averaged over the residues 20 to 28.

Before examining the potential open structures resulting from the different simulations we investigate which combinations of restraints are most effective in producing a stable open pore and examine how the different restraints and tension affect the various functional domains of the protein. Monitoring the pore radius is the most direct structural measure to determine which restraints are most effective in producing an open pore. Figure 3 shows the pore radius as a function of time for some of the simulation protocols. All simulations involving distance restraints (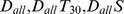 and 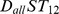) show a steady increase in pore radius during the first half of the simulations in which the restraints are introduced. In contrast, simulations using solvent restraints and/or tension (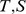 and 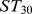) show only a slightly larger pore radius than to simulations without restraints (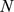). Even simulations using a tension of 30 dynes/cm did not cause spontaneous opening. Previous CG simulations [30] suggest that this is likely a kinetic effect and higher tension is required to observe spontaneous channel opening in the 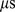 range without restraints. Similar observations were made in previous CG simulations of MscL where tensions above 60 dynes/cm were required to observe a significant pore opening [29].

These results raise the question if the solvent restraints or tension have any effect on the structure when distance restraints are applied. The pore radius of the 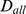 simulation starts to drop off shortly after the restraints are removed while the pore radius of 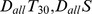 and 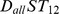 simulations remains more stable, especially for simulations with tension. This suggests that tension and solvent restraints both have a stabilising effect on the open pore structure, something confirmed by comparing the distance restraint energy of the different protocols. A more detailed analysis of the distance restraints can be found in Figure S1 in Text S1.

**Figure 4:**
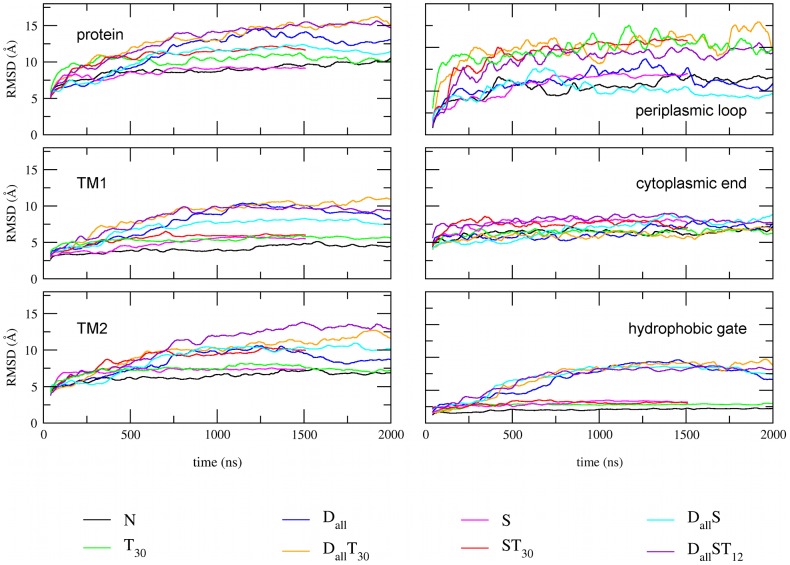
RMSD as a function of time. Comparison of the simulation protocols combining the various types of restraints for the different functional domains of the protein. See also Figure S2 in Text S1.

To investigate the effect of the restraints and tension on the different domains of the protein we prepared plots of root mean square deviation (RMSD) vs time for the various functional domains and simulation protocols (Figure 4 and Figure S2 in Text S1). The RMSD of the hydrophobic gate and TM1 show the same pattern as seen in Figure 3 since the pore radius is calculated from residues in the hydrophobic gate, which is part of TM1. All the simulations involving tension have a larger RMSD of the periplasmic loop than those without, suggesting that tension induces structural rearrangements in this domain. Results for other domains also indicate that the solvent restraints and tension not only stabilise the open pore but have an effect on the structure of the protein independent of the distance restraints.

### Selecting from potential open pore structures

**Figure 5:**
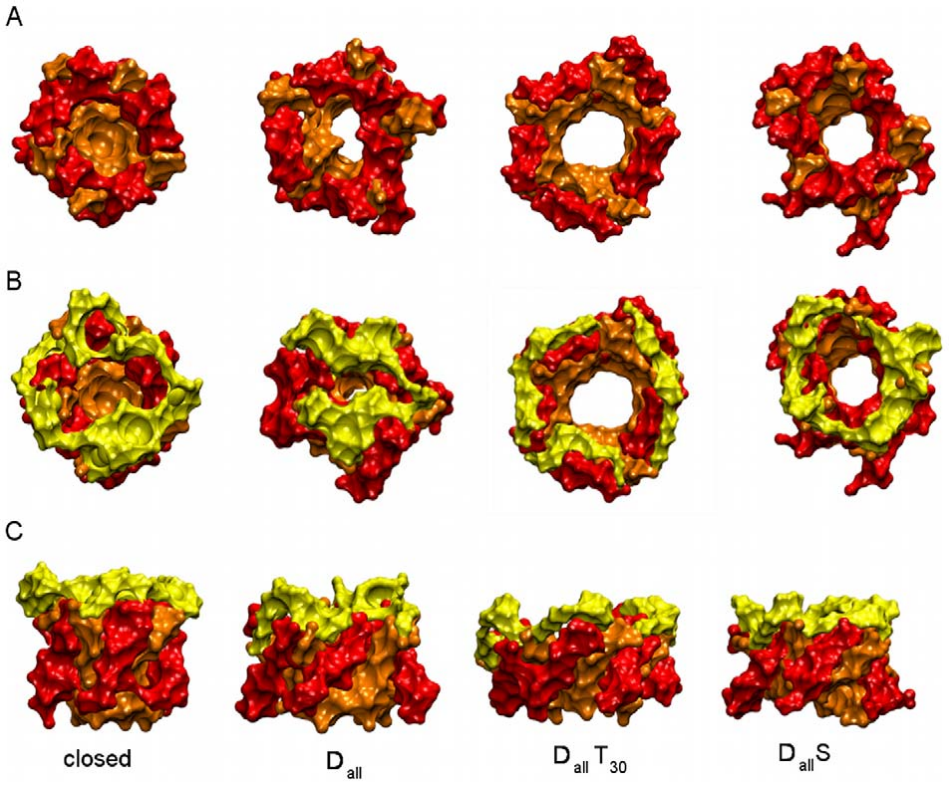
Comparison of potential open pore structures from different simulation protocols represented by the surface of TM1 (orange), TM2 (red) and the periplasmic loop (yellow). For clarity the C- and N-terminals were omitted. A: top view of pore formed by TM1 and TM2. B: top view of pore and periplasmic loop. C: side view of TM1, TM2 and periplasmic loop.

We selected a first set of potential open pore structures from the simulation protocols that produced a structure with a pore radius larger than 12 Å. Comparison of structures from 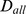 and 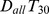 simulations indicates that the pore size at the hydrophobic gate is a necessary but not sufficient structural change to produce an open pore, as can be seen in Figure 5. The structures from 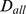 simulations show a widened pore in the TM region but the periplasmic loop failed to expand, occluding the extracellular side of the pore. In comparison, the periplasmic loop from the 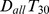 simulation has expanded along with the TM domains. This is also visible from the RMSD vs time plots (Figure 4) which show that the tension increases the extent of structural changes of the periplasmic loop. Analysis of structures from 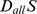 simulations showed that solvent restraints have some effect on the position of the periplasmic loop such that it is not occluding the pore entrance like in the 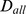 simulation. A more detailed analysis showed that in simulations with tension the periplasmic loop lies more flat on the membrane surface and has a more pronounced tendency to become integrated into pore lining than in the absence of tension. Close inspection of the structures from 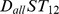 (not shown) revealed that the integrity of the pore is compromised. No such effects were observed in other simulations suggesting that combining the full set of solvent and distance restraints with tension is exerting too much force on the protein. After this visual inspection structures from 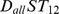 simulations were rejected and not considered for further analysis.

### Comparison of open pore structures

**Figure 6:**
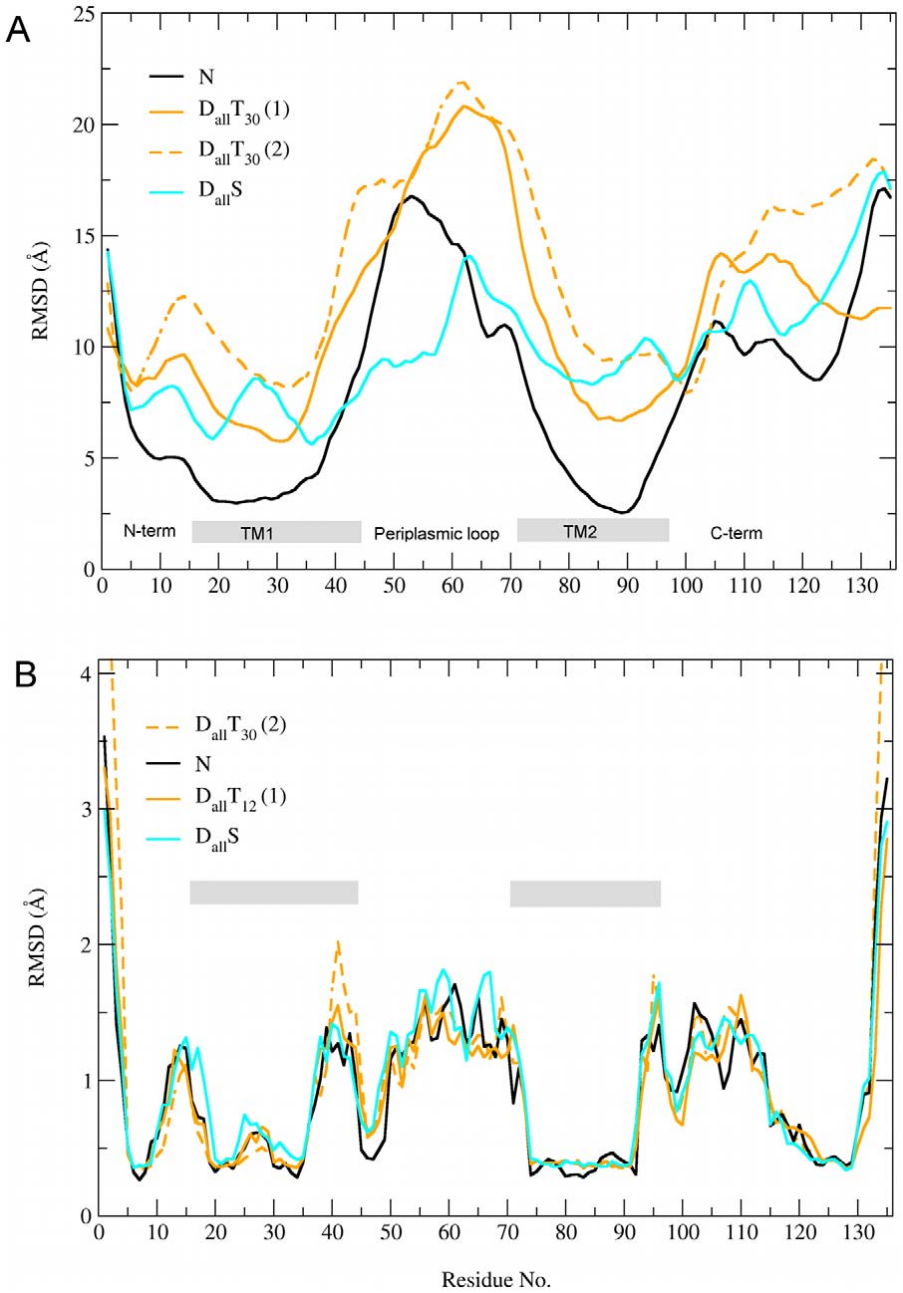
RMSD vs residue for the three open pore structure produced by simulations with distance restraints and tension (dashed and solid orange lines) and simulations with distance and solvent restraints (blue line) in comparison to a structure from a simulation without restraints (black line). Residues corresponding to TM1 and TM2 are indicated by vertical bars. A: RMSD was calculated by aligning the entire protein to the equilibrated closed pore structure. B: RMSD was calculated by aligning consecutive segments of 5 residues to the equilibrated closed pore structure.

Figure 6 shows plots of RMSD vs residue for three open pore structures from 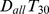 and 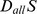 simulations in comparison to a simulation without restraints (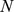). The graphs in Figure 6A were produced by aligning the entire protein of the open pore structure to the equilibrated closed pore structure. The overall pattern of the graphs is very similar to the ones reported in previous MD simulations of MscL [22], [48]. Two structures from simulations with tension (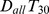) show an increase in RMSD in the periplasmic loop in comparison to the 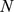 simulation confirming the effect seen in RMSD vs time plots (Figure 4). The increased RMSD suggests that this domain of the protein undergoes more pronounced structural changes in the presence of tension. The increased RMSD of TM1 and TM2 compared to 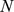 simulations is consistent with an increased mobility of most residues in TM1 and selected residues in TM2 in the open state reported by EPR experiments [7].

To differentiate between rigid body movements of domains and changes in the internal structure of these domains, we prepared plots of RMSD vs residue where the RMSD was calculated by aligning the open and closed structures using consecutive segments of 5 residues (Figure 6B). The data shows that the majority of residues in TM1 and TM2 show low RMSD compared to other functional domains suggesting that the helices maintain structural integrity as may be expected given the bias to maintain secondary structure in the MARTINI force field. However, residues corresponding to the periplasmic end of TM1 (residue 38–43) and the cytoplasmic half of TM2 (residues 90–107) show levels of RMSD as high as the periplasmic loop and the C-terminal. That this arises in all open pore models is indicative that some deformation of the TM helices occurs during channel gating which is discussed in more detail below.

The most distinctive features of the open channel are the pore radius and the tilting of TM1 and TM2, making these structural measurements an obvious choice for comparing our open pore structures to previously reported models of the open channel. Table 3 lists the pore radius and the helix tilt for TM1 and TM2 for the three open structures from this study along with data from previous studies. The pore radius of the 3 structures, calculated as an average over the last 50 ns of simulation, ranges from 13.7 Å to 15.8 Å and is in good agreement with data from EPR experiments (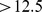 Å) [7], FRET experiments (radius change of 8.0–8.5 Å) [17], estimates based on conductance (15–18.5 Å) [8], [20] and recent atomistic MD simulations (12.0–14.5 Å) [9] and CG simulations (11.6 Å, minimum radius) [30]. Previous unrestrained CG simulations of Tb-MscL at higher tension (77 dynes/cm, 323 K) produced a significantly smaller pore of 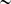
4 Å in radius and an only slightly larger pore of 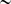
6 Å [29] at increased temperature (338 K) suggesting that combining tension with restraints is more effective that simply increasing the tension in the bilayer. The helix tilt values from simulations without tension (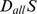) are very close to the values from atomistic MD simulations [9]. Furthermore, both results from this study and from atomistic simulations of MscL [9] predict an increased helix tilt for TM1 and TM2 in the presence of tension. This is consistent with the notion that the helix tilting and the resulting flattening of the pore is a consequence of the protein trying to adapt to the tension-induced change in hydrophobic thickness of the membrane [49] and the suggestion that changes in membrane thickness accelerate the conformational changes involved in the gating process [48]. To estimate the relative motion of the helix pairs that form the pore we calculated the RMSD between the open and closed state by aligning TM1 and TM2 of the same subunit as well as TM1 of one subunit with TM2 of the neighbouring subunit (Table 4). RMSD from TM helices of neighbouring subunits is lower then for TM helices of the same subunit and much lower than from aligning the entire TM domain, suggesting adjacent TM1 and TM2 helices show little relative motion during the gating.

**Table 3:** Pore radius and helix tilt of TM1 and TM2 for selected open pore structures in comparison to previously reported models.

		Helix tilt	
Structure	pore radius ( 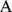 )	TM1 (  )	TM2 (  )
closed	8.5	42	28
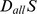	14.3	51	41
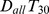 (1)	13.7	62	43
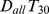 (2)	15.8	67	53
Corry [9] (no tension) (Eco-MscL)	12.0	52	39
Corry [9] (5 dynes/cm) (Eco-MscL)	11.2	56	42
Corry [9] (60 dynes/cm) (Eco-MscL)	14.5	60	47
Sukharev [21] (Tb-MscL)	15.0–18.5	71	46
Meyer [38] (Eco-MscL)	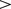 12.5	51.2	54.5
Louhivuori [30] (67 dynes/cm) (Tb-MscL)	11.6 a		

a minimal radius.

**Table 4:** RMSD between equilibrated closed and open pore structures from different simulation protocols.

Structure	RMSD			
	protein	all TM domains	TM1/TM2 same subunit	TM1/TM2 neighbouring subunits
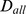	9.6	6.9	4.4	3.5
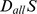	10.7	7.5	5.1	3.4 (5.33) a
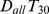 (1)	14.8	11.0	6.8	3.6
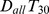 (2)	12.7	8.2	5.7	3.5

RMSD protein and RMSD domain were determined by aligning either the entire protein or all TM helices in the closed to the open pore structure for different sections of the protein.

Values were calculated by aligning each of the specified sections to the equivalent section in the closed state.

a One of the subunits in the 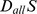 structure shows some deformation in the helix which distorts the average and was not included in calculating the average RMSD. The number in the bracket is the average calculating using all 5 subunits.

**Figure 7:**
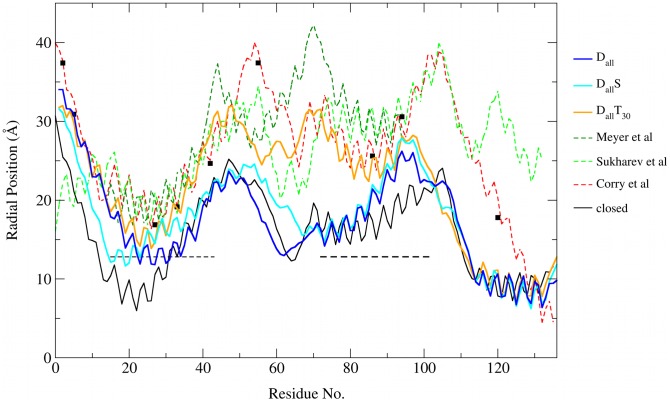
Comparison of open pore structures from this study to previous open-channel models. The lower bound of the EPR distance restraints and the FRET distance restraints are shown as dotted black lines and black squares, respectively.

Figure 7 shows the radial position of each residue for the structures shown in Figure 5, to visualise the structural change involved in gating and to compare these structures to previous open state models. In addition, the plot shows the distance restraints from FRET and EPR. The radial position for the same structures as in Figure 5 are shown as these are the structures that show a pore radius above 12 Å. A comparison of the 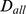, 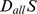 and 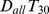 structures to the closed pore confirms that tension is essential to obtain a fully open pore as seen in the much smaller radial position in the TM2 and loop domain in structures without tension, despite an opening in the hydrophobic gate. This confirms the observations from the results in Figure 5. Comparing the 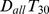 and 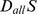 structures to open pore models from previous studies shows a smaller radial position of the residues in the hydrophobic gate. At the same time the pore radius from these structures is in close agreement with these other models (Table 3). This suggests that the same sized pore can be reached with less movement of the backbone. The residues in TM2 from our fully open pore 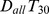 also shows less movement than earlier models. Our results support the notion that a stable and wide pore can be obtained with less structural changes in TM1 and TM2 than suggested by earlier models, as has been noted by Corry et al [9]. Furthermore, there is a marked difference between the C-terminal in our models and previous ones as the radial position remains unchanged in comparison to the closed pore.

**Figure 8:**
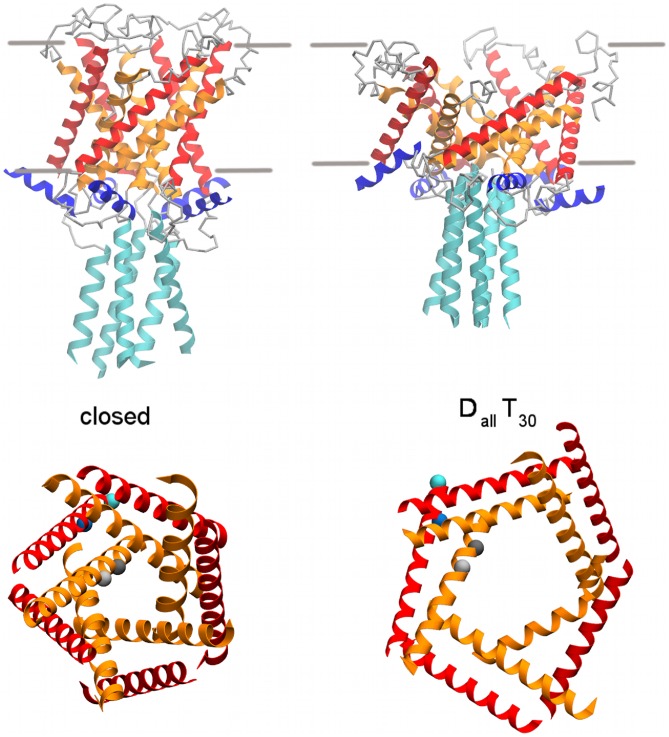
Comparison of the equilibrated closed pore structure to the open pores structures from a simulation with distance restraints and tension (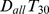). Side view showing the position of the C-terminal and N-terminal. Top view showing the backbone particles of residues V23 (dark grey), G26 (light grey), I92 (blue) and I96 (cyan) that were used as an indication of orientation of TM1 and TM2. TM1 is shown in orange, TM2 in red, the C-terminal in cyan, N-terminal in blue and connecting loops in grey. Structures from other simulation protocols can be found in Figure S3 and S4 in Text S1.

While the distance restraints are based on five-fold symmetry of the closed pore structure, the use of half and flat harmonic potentials for the implementation of the distance restraints means that the symmetry was not rigorously enforced during the simulation. As visible in Figure 8 (as well as Figure S3 and S4 in Text S1) the open pore structures show some loss of symmetry. Based on the data from FRET and EPR experiments on fully labelled MscL proteins it is not possible to tell if and how much the pentameric symmetry is retained during gating. Furthermore, recent unrestrained CG simulations of MscL [30] suggested that the gating mechanism might be asymmetric. We therefore did not enforce or maintain the symmetry during the simulation and did not symmetrize the open pore structures. The structures from the 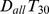 and 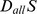 simulations are available as part of the supplementary material (Dataset S1).

### Rotation of TM helices

While there is a general consensus on the iris-like opening and the helix tilting mode of gating there has been little agreement on the potential rotation of the TM helices. The open pore model by Sukharev et al. [20], [21] proposes a clockwise rotation of TM1 while the model reported by Perozo et al. [7], [19] suggested a counterclockwise rotation which was supported by other experimental studies [50], [51]. A similar interpretation was made of results obtained by modifying electrostatic interactions and cross-linking [52]. In contrast, the open pore model obtained from restrained atomistic MD simulations [9] and results from a disulphide cross-linking study [53] showed little rotation of TM1 and no rotation is seen in the crystal structure of the tetrameric *Staphylococcus aureus* MscL (Sa-MscL) in an expanded intermediate state [54].

Figure 8 depicts the equilibrated closed pore structure in comparison to the open pore structure obtained here from a simulation with distance restraints and tension. The open pore structure from other simulation protocols can be found in Figure S4 in Text S1. Using the position of the residues V23, G26, I92 and I96 relative to the pore centre as an indication of TM1 rotation we note that the majority of the residues showed no change in orientation between the closed and open states. Thus, there is little evidence of rotation in the TM helices independent of the restraints used. The lack of rotation and the low RMSD of TM helices from neighbouring subunits indicates that the pair of helices move radially and tilt as a rigid body in agreement with the results of a recent study of the open MscL structure determined by FRET spectroscopy and all atom MD simulations [9]. Note that rotation refers to the change in relative position of the helices as a whole and does not involve changes in secondary structure and hence is not affected by the bias of the MARTINI force field towards maintaining secondary structure.

### Position of C- and N-terminal

In addition to the structure of the pore there has been considerable attention to the structure and possible function of the C- and the N-terminal domains [12], [13], [16], [22], [55]–[57]. An earlier model of MscL suggested that the N-terminal acts as a second gate by forming a helical bundle that occludes the pore on the cytoplasmic side [20], [21], [53]. However, a recently re-examined structure of the Tb-MscL in the closed state [58] and a systematic study of the N-terminal domain [57] shows that the N-terminal forms a helical structure running along the membrane water interface. In all our simulations, independent of pore opening or restraints applied, the N-terminal remained stable, and lies parallel to the cytoplasmic membrane (Figure 8 and S3 in Text S1). The same behaviour was observed in atomistic [9] and coarse grained [30] MD simulations of MscL. The combined results from experimental and simulation studies make it unlikely for the N-terminal to act as a second gate.

In the original crystal structure of Tb-MscL [5] the C-terminal was solved to R118 (corresponding to R126 in Eco-MscL) and the remaining residues were modelled as a continued 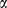-helix. However, the conformation of C-terminal was unusual as a number of hydrophobic residues were pointing outwards to face the aqueous cytosol while some of the charged residues where pointing inside the helical bundle. It has been suggested [59] that this is an artefact of the low pH and the presence of detergent molecules during crystallisation. MD simulations [22], [59] and a re-examined structure of Tb-MscL [58] suggest a more reasonable conformation with the charged residues pointing outwards. The starting structure in our simulations was a homology model of Eco-MscL with a C-terminal similar to the revised Tb-MscL with L121 and L128 facing outwards and L122, I125 and L129 facing inwards. After a 10 ns equilibration without any restraints or tension the orientations of the already inward facing I125, L122 and L129 did not change significantly while L121 and L128 had a tendency to move towards an inward facing conformation. Comparison of the equilibrated and the final structures from different simulation protocols showed that the orientation of the side chains did not change significantly during the 2 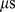 simulations and that there is no difference between closed and equilibrated open pore structures in this respect. Based on our simulations it is likely that these hydrophobic side chains are pointing inside the helical bundle.

In addition to the conformation of the C-terminal there has also been a different hypotheses as to the functional role of the C-terminal. Some studies suggested that the C-terminal dissociates during opening of the pore [8], [54], [60] while others suggested it might act as a size-exclusion filter [59]. As depicted in Figure 8 and Figure S3 in Text S1 the helical bundle remained intact in all our simulations which is consistent with results from cross-linking [59] and EPR [38], [61] experiments. Comparison of the structures from different simulations revealed an upward movement of the C-terminal that was present in all simulations independent of restraints or tension. Figure 8 depicts the position of the C-terminal in the equilibrated closed pore in comparison to the open pore structure from a simulation with distance restraints and tension. Structures from other simulations can be found in Figure S3 in Text S1. The upward motion of the C-terminal results in contacts between the C-terminal and transmembrane domains.

### Structural changes induced by tension

A number of results from the different analyses point towards the role of tension in structural changes during gating. Visual comparison of the open and closed pore structures revealed a change in position of the periplasmic loop as well as structural rearrangements in the upper part of TM1. In the closed pore the periplasmic loop tends to point towards the extracellular space. This is in contrast to the open pore in which the loop shows an outward motion towards the membrane surface. Comparison of structures from different protocols revealed that this structural change is much more pronounced in simulations with tension. In the absence of tension the loop mostly remains in the extracellular space and often occludes the pore entrance while tension causes the loop to move towards the membrane surface and away from the pore centre. This conformational change of the loop is not correlated with pore opening at the hydrophobic gate. 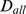 simulations resulted in a channel with an increased pore radius at the hydrophobic gate but the loop showed no outward motion, causing the loop to occlude the pore entrance (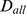 in Figure 5). In contrast, 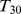 and 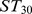 simulations did not induce opening at the hydrophobic gate but the loop moved towards the membrane surface resulting in an accessible pore. These observations imply that tension is strongly related to the outward motion of the periplasmic loop during gating and that this is independent of the opening at the hydrophobic gate. Similar observation were reported in a recent study using MD simulations to investigate the energetic contributions to MscL gating [32] showing a tension-dependent increase of area on the extracellular side of the protein even when the hydrophobic gate remains closed. The results from this study further showed that a large part of the gating energy is actually provided by the adaptation of the channel at the lipid-water interface.

Further inspection of the open pore structures showed that the re-orientation of the periplasmic loop is accompanied by the formation of a kink in the upper part of TM1 (around residue 40) (see also [62]). The formation of the kink is only possible and might be induced by the simultaneous thinning of the membrane as a straight helix is required to span the resting thickness of the membrane. In contrast, the kink allows the top of the helix to lie along the surface of the thinned membrane.

To further investigate the formation of the kink in simulations with and without tension we first carried out a visual inspection of the average structures from the different simulation protocols to identify the presence of a kink and its position. Figure S5 in Text S1 summarises the results by showing the fraction of structures for which a visible kink is present as well as the position of the kink for simulations in POPC without tension, simulations in POPC with tension and simulations in DMPC without tension. In simulations without tension the kink occurs outside of TM1 while structures from simulations with tension show a shift in the position, with the majority of kinks occurring between residues 38 to 44, corresponding to the periplasmic end of TM1. This effect is also visible in Figure 6 that revealed a change in the internal structure of the upper part of TM1 around residues 37 to 45. The combined results support the observation that the formation of the kink and the concurrent outward movement of the periplasmic loop during gating are related to presence of tension in the membrane. To further support the hypothesis that there is an increased occurrence of kinks in the upper part of TM1 in the presence of tension we carried out a more quantitative analysis where the kink was defined as the deviation from a straight helix as found in the closed pore structure. An RMSD vs residue was calculated for the TM1 helices in the 17 structures by comparing them to the close pore. Based on these datasets we calculated the % occurrence of kinks for simulations with and without tension. 39% of subunits from simulations without tension showed a deformation of the external end of TM1 compared to the closed structure and this number increased to 51% in simulations with tension. Based on these findings we hypothesise that the thinning of the membrane is associated with the formation of a kink in the upper part of TM1 and that this structural change is accompanied by an outward motion of the periplasmic loop towards the membrane surface.

### Simulations of MscL in short-chain lipids

To support our hypothesis regarding the effect of tension on the periplasmic loop we conducted simulations of the MscL embedded in DMPC, a lipid that yields a thinner membrane in comparison to POPC without the application of tension. The pore radius of two final structures from DMPC simulations is 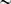
8.5 Å, which is almost equal to the pore radius of the equilibrated closed pore. That the hydrophobic gate is closed is further confirmed by a nearly constant RMSD for residues in the hydrophobic gate (Figure S6 in Text S1). There is a clear increase of RMSD of the periplasmic loop in DMPC simulations to the same level as seen in POPC 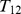 and POPC 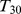 simulations indicating that the periplasmic loop responds to the thinner membrane in the same way as it responds to tension. This increased activity of the periplasmic loop is also seen in plots of RMSD vs residue for DMPC and POPC simulations (Figure S7 in Text S1). The results confirm that the periplasmic loop exhibits similar behaviour in DMPC simulations as in POPC 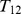 simulations with tension.

Apart from a small increase in RMSD in TM1 there are no significant structural changes in the other functional domains of the protein. None of the characteristics of the open pore such as an increase in pore radius and helix tilt were observed in DMPC simulations further supporting the notion that the activity in the periplasmic loop is independent of channel gating. We repeated the analysis to identify the position of the kink for structures from DMPC simulations. Figure S5 in Text S1 demonstrates that there is an increased occurrence of kinks around residues 39 to 41 in DMPC simulations. Our quantitative analysis showed that 44% of subunits from the DMPC simulations showed a kink. It is worth emphasising that these simulations did not employ any restraints or tension. Hence the thinning of the membrane in DMPC was enough to induce the formation of kinks similar to simulations in POPC with tension, independent of other major structural changes and pore opening.

## Discussion

The aim of this study was to model the open pore structure of the MscL protein and investigate the structural changes involved in gating using restrained CG MD simulations. The simulations were carried out using a homology model of Eco-MscL [20], [21], [38] instead of the crystal structure of Tb-MscL as the restraints in the simulations are based on data from FRET and EPR experiments that were carried out on Eco-McsL. To model the open pore we integrated solvent accessibility and inter-subunit distances from EPR and FRET experiments into a CG model of the closed state of MscL and carried out a series of simulations using different combinations of restraints and membrane tension. The use of a CG model allowed us to run multiple simulations in the 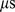 range and observe structural changes not seen in single shorter simulations. Furthermore, combining the CG model with restraints allowed for much greater conformational sampling than has previously been possible. While previous simulations used either large tensions or radial forces to induce opening of the pore within the available simulation time, here the opening is achieved through the use of restraints based on experimental data. While this also involves the application of external forces that move the protein towards a predetermined goal and which can distort the protein if the biasing forces are applied too quickly, we hope that these forces at least lead to a conformation that is consistent with the known structural data from experiments. In addition, the long simulation time allowed us to slowly introduce the restraints, apply tension that is of the same magnitude as the physiological tension of MscL as well as keep the force constants of all restrains much lower than in shorter, atomistic simulations. Furthermore, we carried out multiple simulations with different combinations of restraints, testing the sensitivity of the final structure to the restraints used. However, as a consequence of using experimental restraints to direct the evolution of the system through conformational space towards an open pore structure, the simulations should not be used to study intermediate structures or analyse the order of structural changes during gating.

From the restrained simulations we produced a set of plausible open pore structures that showed some common features. The pore radius of the open pore structures ranged from 13.7 Å to 15.8 Å, in good agreement with results from experimental [7], [8], [17], [20] and other simulation studies [9], [30]. Our results suggest that a stable open pore can be achieved with less structural changes than previously reported. The open pore structure showed no significant rotation of the TM helices and adjacent TM1 and TM2 helices move together as fairly rigid unit. The open pores structures showed deviations from the five-fold symmetry of the open state, which was also observed in previous CG simulations [30] that suggested an asymmetric gating mechanism.

Analysis of the final structure from all simulations showed that the N-terminal sits at the cytoplasmic lipid interface making it highly unlikely to act as a second gate. Our results also support that the C-terminal remains as an intact helical bundle and shows no outward motion away from the central axis running down the pore centre. In addition, the outward facing conformation of the hydrophobic residues in the C-terminal are likely to be an artefact of the crystallisation. The simulations did not contain any solvent restraints and only a single distance restraint in the C-terminal. Furthermore, we observed that the C-terminal bundle is free to move and, in comparison to the crystal structure, moves upwards to contact the TM section of the protein. While it could be an artefact of the simulations, this upward movement was observed in all simulations independent of tension and pore opening. It is possible that the low position of the C-terminal in the crystal structure is another artefact of crystal packing and the long simulations allowed this domain to move into a more natural position.

On the other hand, this upward movement might be of functional relevance. The upward movement of the C-terminal reduces the size of the entrance to the pore and is possibly related with the hypothesis that the domain acts as a size exclusion filter [59]. It is also possible that the upward motion reveals interactions between the C-terminal and the TM helices that have not been observed previously. These interactions could stabilise either the open or closed state, depending if the pore is closed or open when the contact arises. In the case of the open state, the contact might prevent the closing of the pore by blocking the movement of the TM helices which might be related to the increased frequency of sub-conducting states in 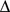
110–136 mutants of MscL [13], [59]. A blocking of the open gate by the C-terminal was also observed in recent coarse grained simulations of MscL [30]. On the contrary, recent results from patch-clamp experiments [54] showed that Sa-MscL C
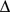
26 reconstituted in liposomes is more stable in the open state than WT channel. The authors suggested that this is because the C-terminal stabilises the closed state and hence the truncated mutant is more likely to sample the open state. The interactions between the C-terminal and the TM helices resulting for the upward movement of the C-terminal could be related to this effect.

One of the most interesting result from our simulations is related to the structural changes of the periplasmic loop. Simulations with membrane thinning, induced by tension or the use of short-chain lipids, showed a significant structural change in the periplasmic loop and an outward motion of the entire loop away from the pore centre such that it lies close to the membrane surface. In contrast, in simulations without membrane thinning the loop remains around the pore entrance on the extracellular side where it tends to partially block this end of the pore. The reason for this structural re-arrangement of the periplasmic loop appears to be the formation of a kink in the upper part of TM1 which allows the upper end of TM1 and the start of the periplasmic loop to move outwards towards the surface of the thinned membrane. Both the outward motion and the formation of the kink are independent of pore opening. Based on these observations we propose that membrane thinning induces a kink at the top of TM1 which results an in outward motion of the periplasmic loop. These previously unobserved structural changes might have important implications as they reveal a new mechanism of sensing and transducing tension by or to the extracellular domains of the protein. This is an alternative to the conventional response to hydrophobic mismatch in which the protein senses membrane thinning by tilting of the TM helices in an attempt to avoid exposure of hydrophobic residues at both ends of the TM helices to aqueous environment. This new mechanism of tension sensing by helix kinking can act in concert with helix tilting and might contribute to the still unanswered question of how MS channels sense tension in the bilayer. Our observations are consistent with the results from a number of other studies. MD simulations of MscL in a curved bilayer showed spontaneous restructuring of the periplasmic loop leading to contacts between the loop and lipid head groups [62]. This contact has also been documented experimentally [38], [63]. EPR experiments showed that, on average, the lipid accessibility of residues in the periplasmic loop increases in the open channel [38]. Cleaving of the loop [56] or mutations [13] in this domain altered the channel's sensitivity to tension. Finally, patch-clamping experiments [64] on WT-MscL and a series of Eco-MscL mutants reported that mutations of residues in the upper part of TM1 show reduced or complete loss of channel activity.

We can propose two different but related functional consequences of the observed structural change of the periplasmic loop. First, the formation of the kink and the outward motion of the periplasmic loop might prevent unwanted or random opening in the absence of tension as it makes it harder for TM helices to move and open the gate unless the periplasmic loop has first extended into the outward position. This proposition is in agreement with experiments that show that the energy of activation is lower in MscL reconstituted in phosphatylcholine (PC) containing 16 carbons and higher in PC with 20 carbons compared to the commonly used POPC with 18 carbons [19]. The results also agree with a recent study showing that introducing lipids of shorter chain lengths to WT-MscL reconstituted in azolectin reduces the tension required to open while adding cholesterol to stiffen the membrane causes an increase in gating tension [64]. It has been suggested previously that the periplasmic loop acts as a “spring that resists the opening of the channel" [56] and our results relate this functional role of the periplasmic loop to specific structural rearrangements of that domain. If the loop resists opening it is likely that this domain is the first to undergo structural changes during gating, as previously suggested [62]. Secondly, in the case of an unwanted opening of the gate in the absence of tension or membrane thinning, the partial blocking of the extracellular end of the pore by the periplasmic loop might prevent the excessive loss of solutes.

The open pore models produced in this study provided us with valuable insight into the structural changes involved in MscL gating and they form a good starting point for further refinement of the open pore model. By running and comparing two simulations for almost all protocols and running simulations with reduced data sets we ensured a basic level of reproducibility. Nevertheless, to further refine the open pore models it would be useful to run multiple simulations using the protocols that proved most effective in producing open pores and perform cluster analysis on a number of open pore structures. A set of representative open pore structures can then be used to carry out atomistic simulations based on the CG model to obtain an atomistic open pore model.

The methodology of combining CG simulations with restraints based on experimental data has enabled us to conduct an extensive analysis of the structural changes of the protein during gating and to find some previously unreported structural changes. This approach is not limited to modelling mechanosensitive ion channels and may be useful to investigate the structure and function of other membrane proteins. The determination of high resolution structures for membrane proteins is challenging, as demonstrated by the low number of membrane protein structures in comparison to soluble proteins in the Protein Databank. Furthermore, for many membrane proteins the available high-resolution structure only represents one conformation but in most ion channels and membrane transport proteins the structure of multiple conformations is needed to elucidate the structure-function relationship. MD simulations that use low resolution, structural data can be very useful to refine the structure of membrane proteins in a given conformation or to model the remaining conformational states. While the integration of geometry restraints such as distances, angles and relative positions is implemented in most MD simulation packages the use of solvent accessibility is not as straight-forward as these restraints require changes to the non-bonded interactions in the force field. Nevertheless, restrained MD simulations are a promising tool to model the structure and conformational changes in membrane proteins.

## Supporting Information

Dataset S1Open pore structure of MscL from a coarse grained simulation using distance and solvent restraints combined with membrane tension (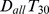 (1)) showing a pore radius of 13.7 Å. Each residue is represented by a backbone particle and 0 or more side chain particles. Open pore structure of MscL from a coarse grained simulation using distance and solvent restraints combined with membrane tension (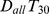 (2)) showing a pore radius of 15.8 Å. Each residue is represented by a backbone particle and 0 or more side chain particles. Open pore structure of MscL from a coarse grained simulation using solvent restraints (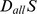) showing a pore radius of 14.3 Å. Each residue is represented by a backbone particle and 0 or more side chain particles.(ZIP)

Text S1 The supplementary material contains additional analysis of the distance restraints (Figure S1), supporting Figures for the structural analysis of the potential open pore structures (S2 to S4) as well as additional data for the analysis of the kink in TM1 (Figure S5) and the simulations of MscL in short-chain lipids (Figure S6 and S7). It also contains the protocols used for the structural analysis of the simulations and a more detailed description of the implementation of distance and solvent restraints (Table S1 to S3).
(PDF)
