# Supplementary material for: Structural Investigation of MscL Gating Using Experimental Data and Coarse Grained MD Simulations
Source: PLoS Comput Biol. 2012 Sep 20;8(9):e1002683. doi: 10.1371/journal.pcbi.1002683 (PMC3447979; doi:10.1371/journal.pcbi.1002683)
Supplement: Text S1 — The supplementary material contains additional analysis of the distance restraints (Figure S1), supporting Figures for the structural analysis of the potential open pore structures (S2 to S4) as well as additional data for the analysis of the kink in TM1 (Figure S5) and the simulations of MscL in short-chain lipids (Figure S6 and S7). It also contains the protocols used for the structural analysis of the simulations and a more detailed description of the implementation of distance and solvent restraints (Table S1 to S3). (PDF) [file pcbi.1002683.s002.pdf]

Structural investigation of MscL gating using experimental data and coarse grained MD simulations - Supplementary Material

Evelyne Deplazes[#], Martti Louhivuori[*], Dylan Jayatilaka[#], Siewert J. Marrink[*] and Ben Corry[#†]

[#] School of Biomedical, Biomolecular and Chemical Sciences,
The University of Western Australia, Perth, Australia

[*] Groningen Biomolecular Sciences and Biotechnology Institute
and Zernike Institute for Advanced Materials,
University of Groningen, Groningen, The Netherlands

[†] Research School of Biology,
The Australian National University, Canberra, Australia

ben.corry@anu.edu.au

Effect of restraints

Plots of pore radius vs simulation time (Figure 3 in main article) demonstrated that the distance restraints are much more effective than the solvent restraints in producing an open pore but also suggested that solvent restraints and tension reinforce the distance restraints and have a stabilising effect on the open pore structure. To further investigate this we analysed the distance restraints as a function of simulation time in the presence and absence of solvent restraints and tension (Figure S1). The data confirms that both EPR and FRET restraints have a strong effect on the protein structure as seen by the rapid drop off in restraint energy in the first 500 ns of simulation. Similar to the results plots of pore radius vs time the data from the first 1500 ns shows some indication that the distance restraints are reinforced by the solvent restraints and tension as the restraint energy drops off faster and to a slightly lower level for simulations involving tension and solvent restraints. Despite this small difference, the restraint energy clearly remains lower for simulations that combine distance restraints with tension and solvent restraints after the restraints are removed at 1500 ns. This supports the stabilising effect of the solvent restraints and tension on the open pore structure. Furthermore, the data shows that FRET restraints re-enforce the EPR distance restraints and also vice-versa. In all simulations the energy of FRET restraints never decreases to the same low level as the EPR restraint energy and FRET restraints show a larger increase when the restraints are removed. This is most likely a consequence of the different implementation of the EPR and FRET restraint energy. The EPR restraints are implemented as a harmonic half-potential while for the FRET restraints a flat harmonic potential was used. In addition, the EPR restraints are exclusively in the TM helices, a domain of the protein that is less mobile. In contrast, the FRET restraints are distributed over the entire protein. Hence it is harder for the system to reach a structure where all FRET restraints are fulfilled.

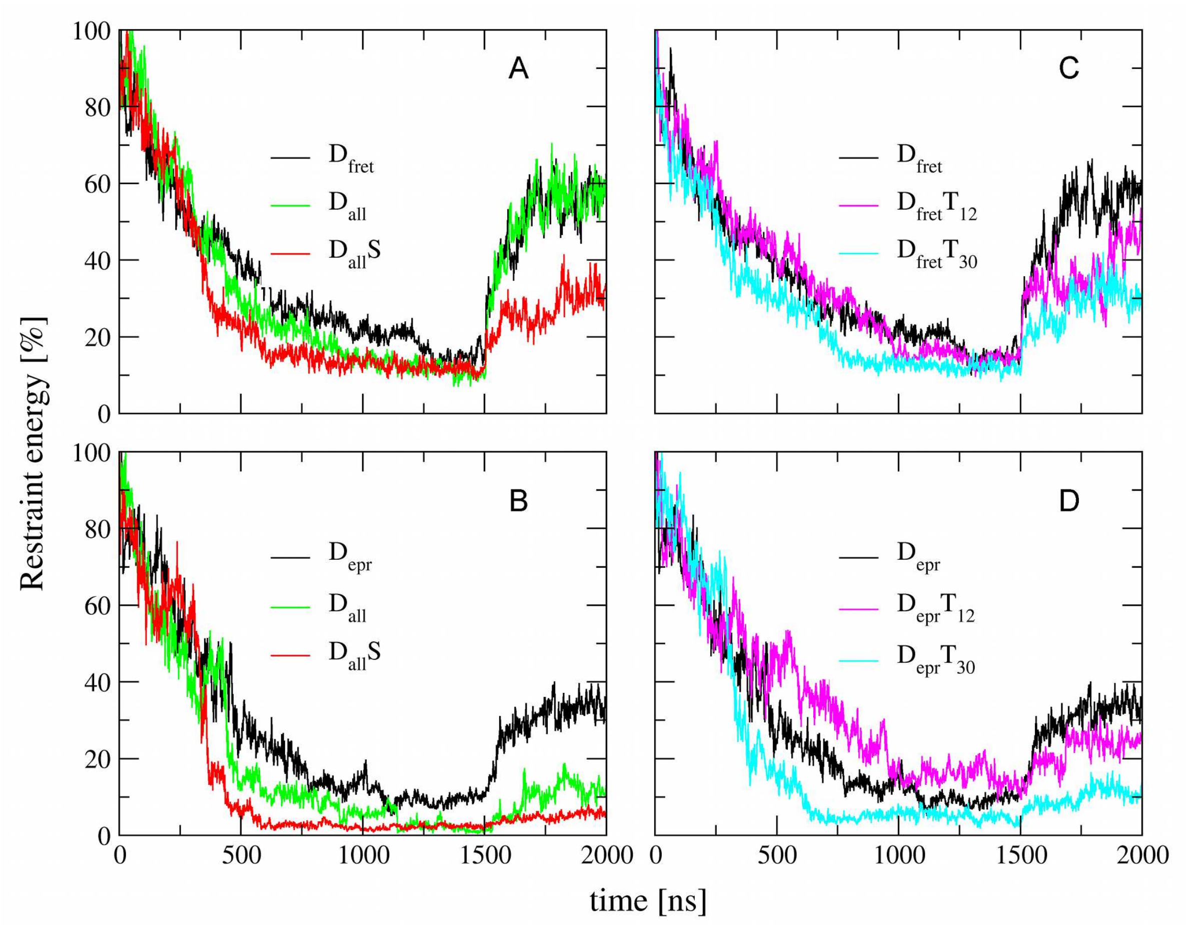

Figure S1: Energy of FRET and EPR distance restraints as a function of simulation time for different simulation protocols. A/B Effect of combining EPR restraints with FRET restraints and solvent restraints. C/D: Effect of tension on distance restraints. The restraint energy is shown as a % of the maximum value at the start of the simulation. Restraints are introduced over 1000 ns, held constant for the next 500 ns and removed during the last 500 ns of the simulation.

RMSD analysis

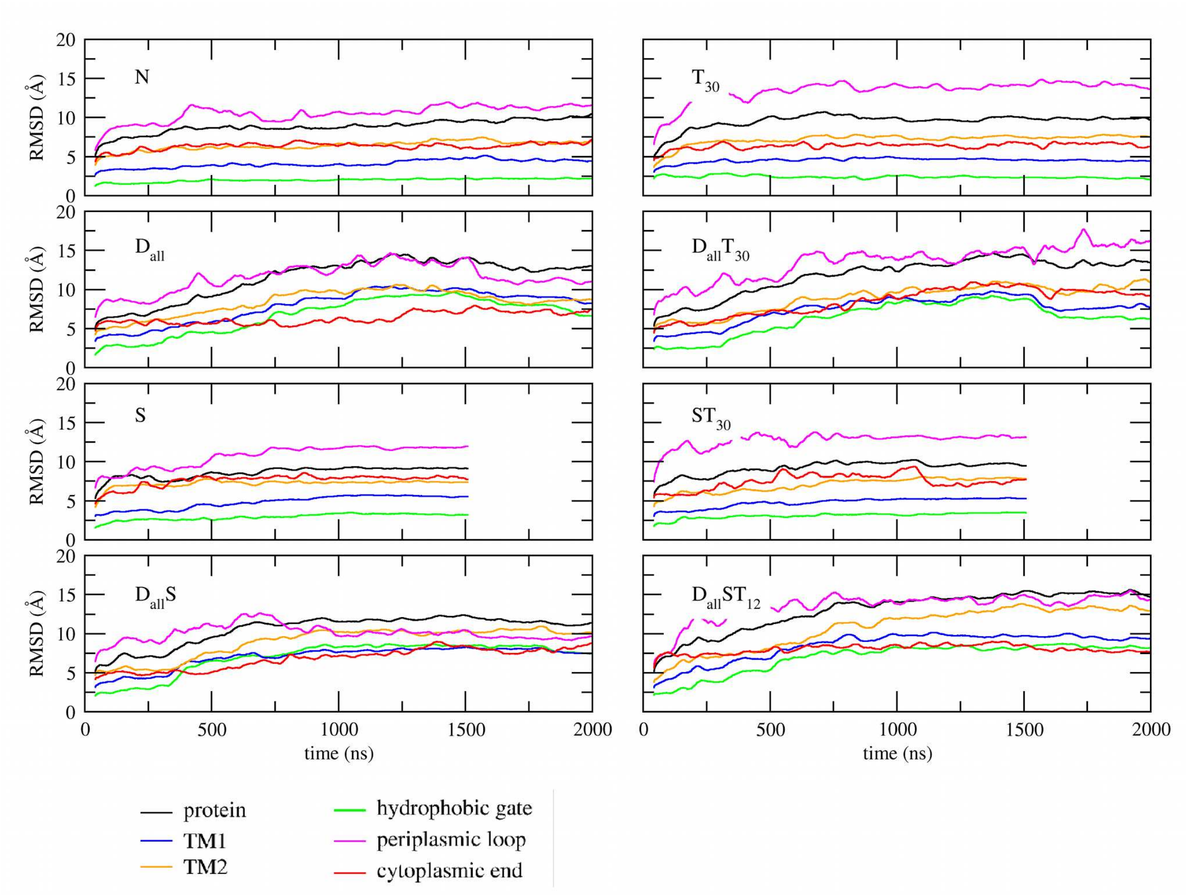

Figure S2: RMSD as a function of simulation time. Comparison of functional domains for the different simulation protocols (related to Figure 3 in the main article).

Position of C- and N-terminal

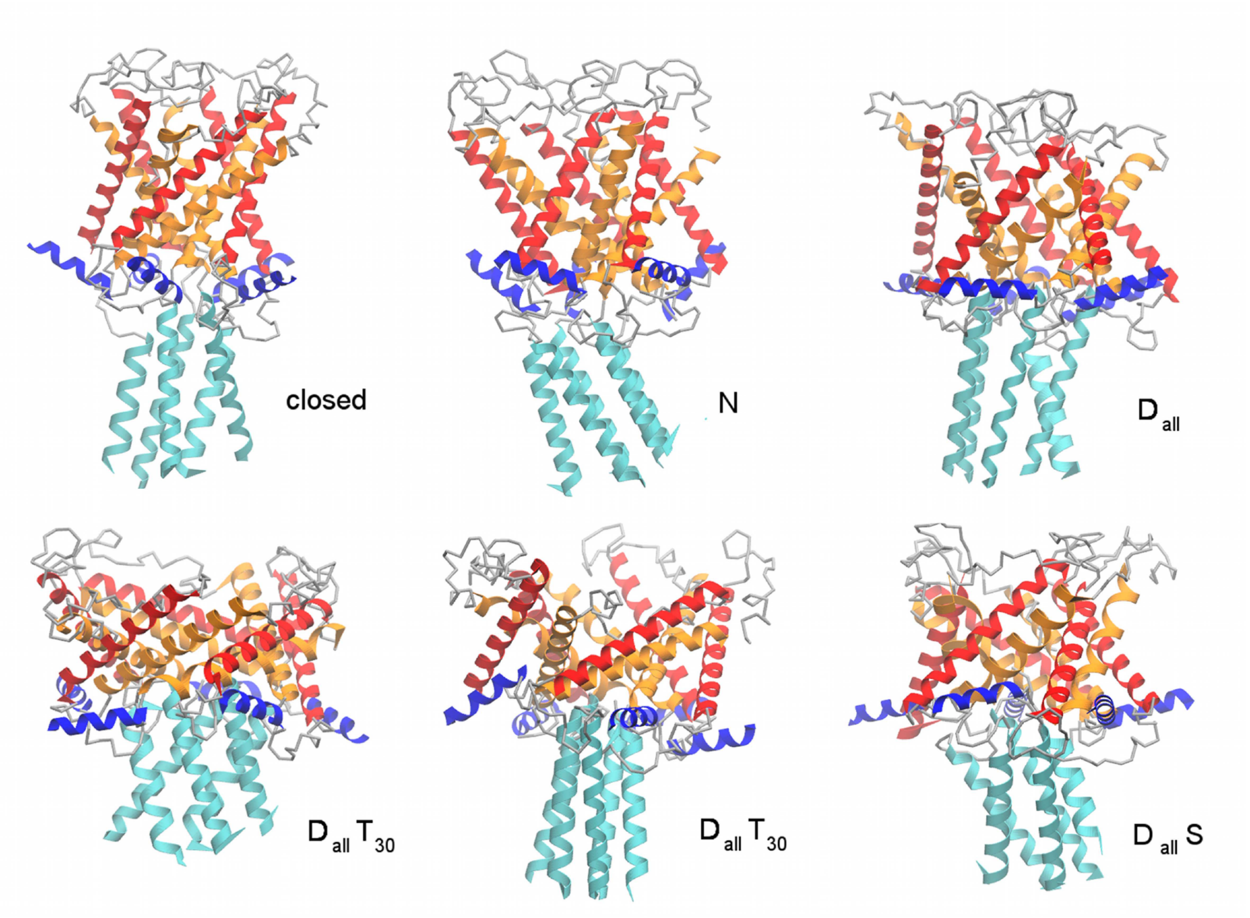

Figure S3: Position of C-terminal in the equilibrated closed pore structure, the final structure from a simulation without restraints (N) and open pore structures from simulations with different combinations of restraints. TM1 is shown in orange, TM2 in red, the C-terminal in cyan, the N-terminal in blue and connecting loops in grey.

Rotation of TM helices

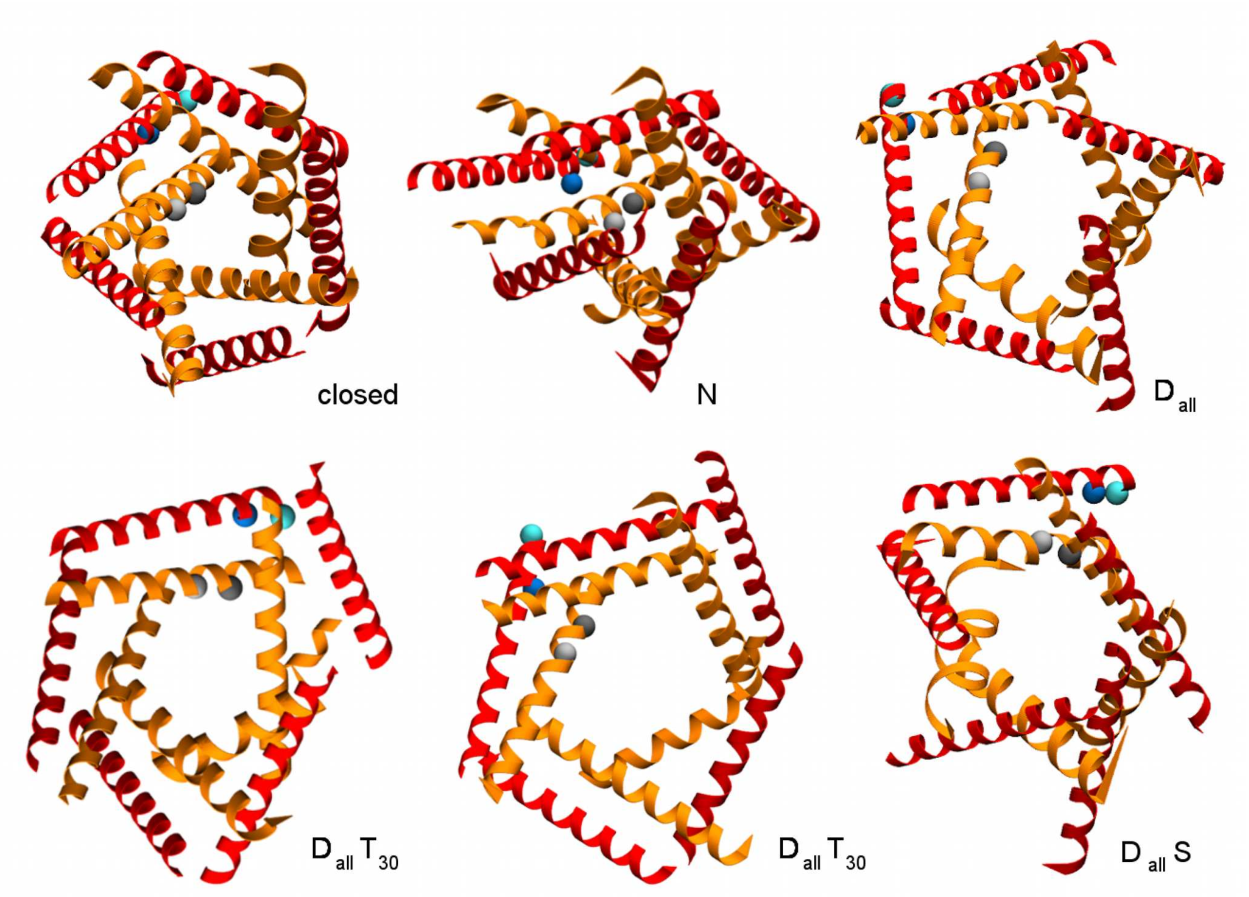

Figure S4: Top view of structures from the the equilibrated closed pore, the final structure from a simulation without restraints (N) and open pore structures from simulations with different combinations of restraints. TM1 is shown in orange, TM2 in red. Backbone particles of residues V23 (dark grey), G26 (light grey), I92 (blue) and I96 (cyan) were used as an indication of orientation of TM1 and TM2.

Results from kink analysis

A first analysis of the formation of a kink in the upper part of TM1 was carried out using a visual inspection of 17 structures from different simulation protocols to identify the presence of a kink and its position. Figure S5 summarises the results by showing the fraction of structures for which a visible kink is present as well as the position of the kink for simulations in POPC without tension, simulations in POPC with tension and simulations in DMPC without tension.

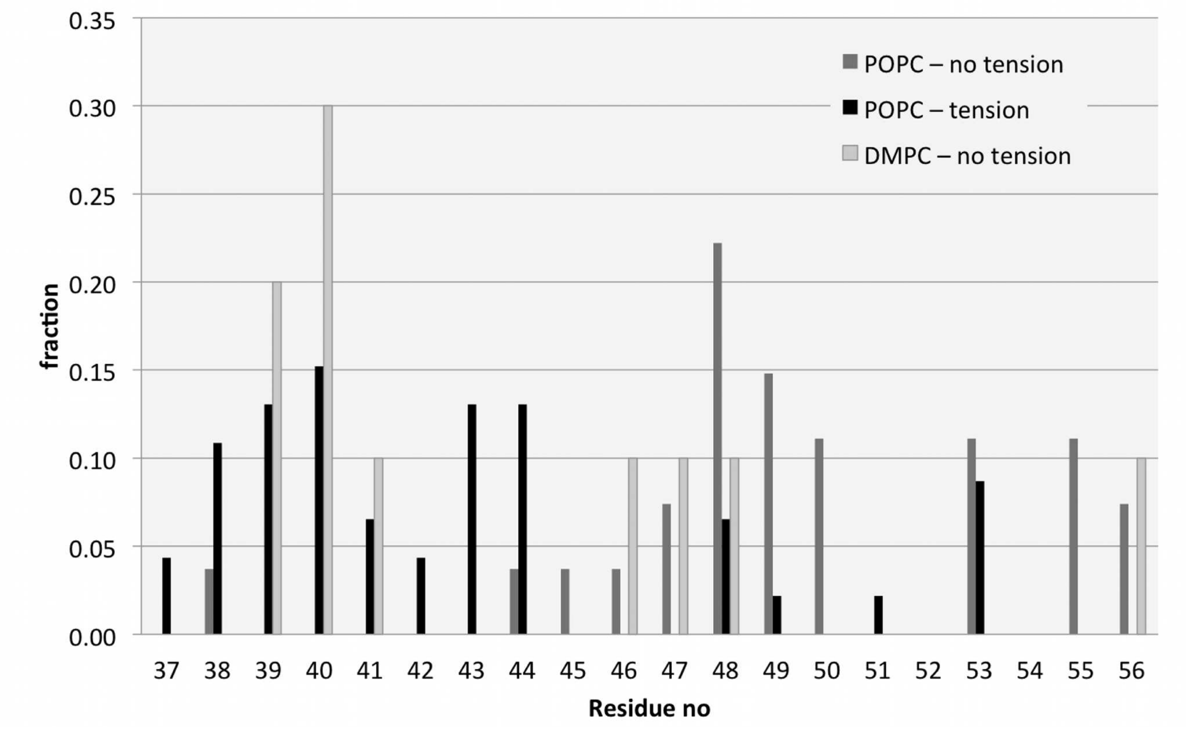

Figure S5: Histogram summarising the position of a kink in TM1 for restrained simulations in POPC (with and without tension) and unrestrained simulations DMPC (without tension). fraction is the proportion of the structures under each condition for which a visible kink is present.

Results from simulations in short chain lipids

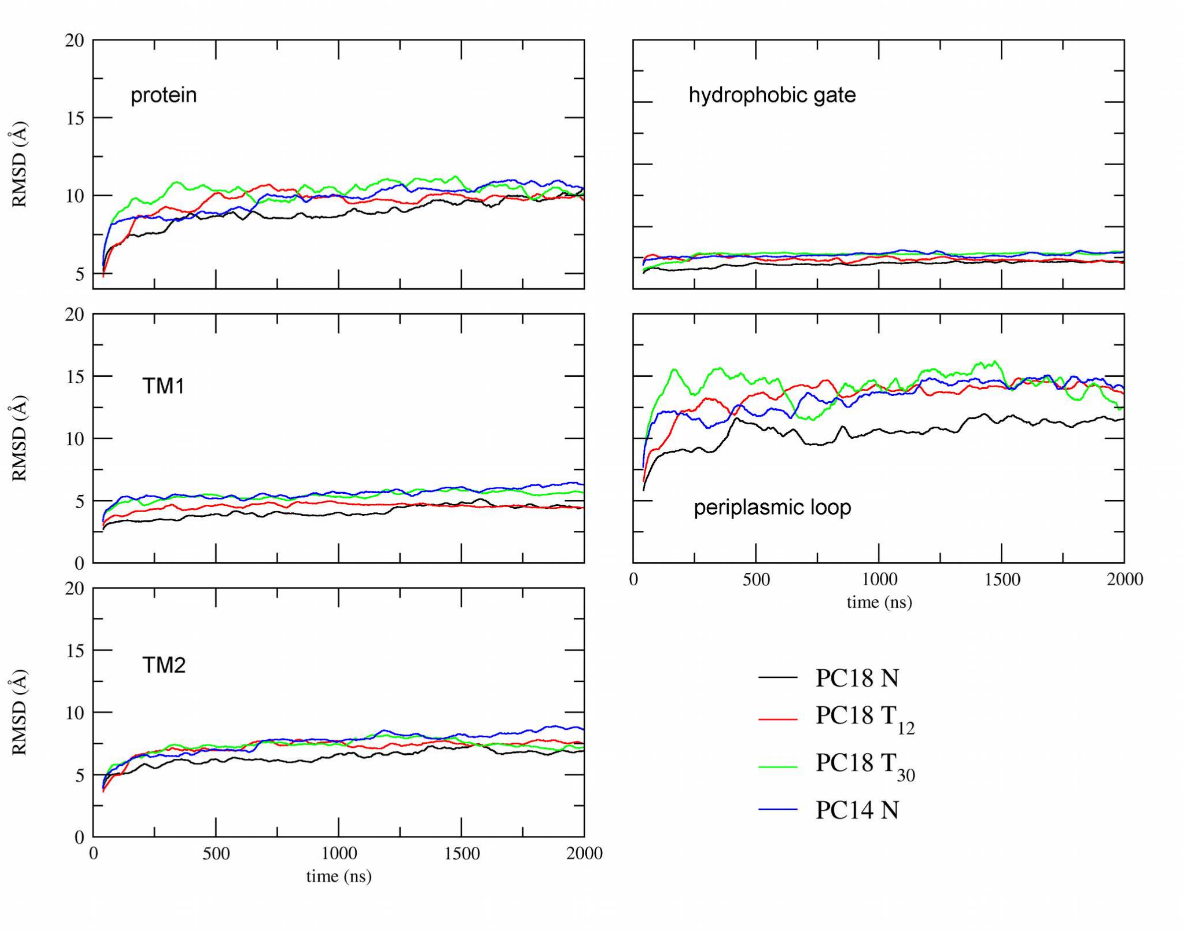

Figure S6: RMSD as a function of simulation time. Comparison of DMPC and POPC simulations for different functional domains of the protein.

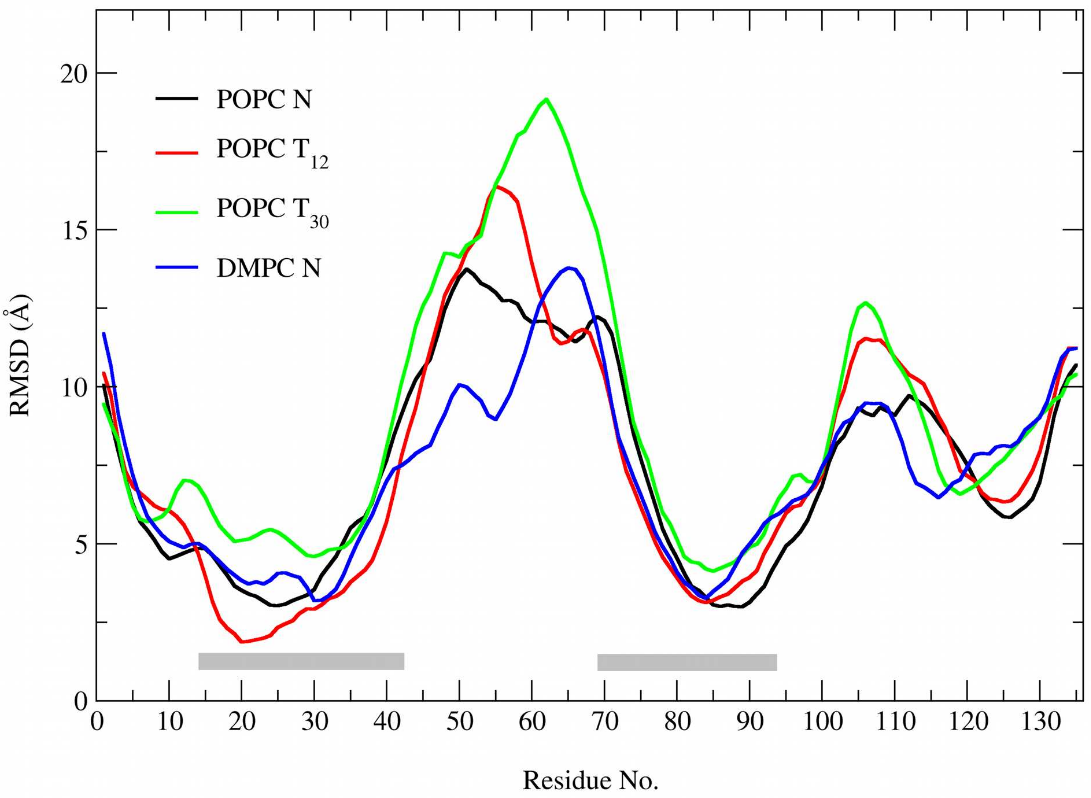

Figure S7: RMSD vs Residue for DMPC and POPC simulations. RMSD was calculated by aligning the entire protein to the equilibrated closed pore structure.

Protocol for structural analysis of simulations

Distance Restraints: The energy of the EPR and FRET distance restraints was calculated as a means of testing if the distance restraints are being fulfilled and to investigate the effect of combining distance restraints with solvent restraints or tension. The energy of the restraints was calculated from the difference between the target distance (d_{open}) and the instantaneous distance (r_{ij}) between the restrained residues using the potential defined in Eq. 2 and Eq. 3 and summed up over all restrained residues.

Pore radius: The most characteristic feature of the open pore structure is the widening of the hydrophobic gate formed by the backbone particles residues 20 to 28 in TM1. The pore size was estimated by the radius of the circle inscribing the pentagon formed by the 5 subunits and averaged over the residues 20 to 28. The pore radius of open pore structures was determined by averaging the radius calculated from 200 frames in the last 50 ns of the simulations.

Helix tilt: The helix tilt for TM1 and TM2 was estimated by calculating the angle formed by the helix and the normal to the membrane plane. To calculate the helix tilt the direction of TM1 and TM2 were estimated by an axis passing through the backbone particles in each TM helix. The TM1 and TM2 helix tilt was averaged over the 5 subunits and calculated for each frame in the trajectory. The helix tilt for an open pore structure was calculated by determining the average angle of TM1 and TM2 determined by averaging the angles calculated from 200 frames in the last 50 ns of the simulation.

RMSD of functional domains: To monitor the effect of the various combinations of restraints on the protein, the RMSD between the equilibrated closed structure and the structure at each time step in the simulation was calculated. Plots of RMSD vs time were prepared for the entire protein and the following functional domains: i) the hydrophobic gate formed by residues 20 to 28, ii) transmembrane helices TM1 and TM2 formed by residues 15 to 43 and 72 to 107, iii) the periplasmic loop consisting of residues 44 to 71 and iv) C-terminal formed by 108 to 136.

RMSD between structures: The RMSD between the closed equilibrated and selected open pore structures was determined by averaging the RMSD values for the specified sections from a RMSD vs time plot calculated from 200 frames in the last 50 ns in the simulation. To ensure the per-residue RMSD is converged the data from the last 50 ns was compared to data sets obtained from 200 frames between 1100 - 1150 ns and 1600 - 1650 ns.

RMSD vs residue: RMSD vs residue was calculated for selected open pore structures based on the RMSD between the equilibrated closed structure and the open structure by averaging the RMSD between closed and open structure for each residue from 200 frames in the last 50 ns of the simulation. RMSD was calculated by either aligning the entire protein or consecutive segments of 5 residues.

Radial position vs residue: The radial position was used as measure of how far a residue

has moved outwards with respect to the pore centre during the simulation and was calculated as the distance between the backbone particle and the pore centre, averaged over 200 frames from the last 50 ns of the simulation. The radial position for each residue was averaged over the 5 subunits. To compare our open pore structures to models from previous studies we calculated the radial position of the α-Carbon in each residue for structures from Sukharev et al [1, 2] , Meyer et al [3] and a model from atomistic restrained MD simulations by Corry et al [4].

Kink analysis The kink analysis was carried out on 17 structures from different simulation protocols. The structures were divided into 3 groups; 7 structures from simulations without tension in POPC (with a variety of distance and solvent restraints), 7 structures from simulations with tension in POPC (wit a variety of distance and solvent restraints) and 3 structures from simulations in DMPC. The first to identify the presence of a kink and its position was based on the visual inspection of the average structure calculated from the last 50 ns of each of the different simulations protocols. To confirm our hypothesis we carried out a quantitative analysis that did not use any average structures to remove any potential artefacts caused by the averaging of multiple structures. In the quantitative analysis the kink was defined a RMS deviation from a straight helix as found in the closed pore structure. For each of the 17 structures 25 frames from the last 50 ns of the simulation were analysed using the TM1 helices from the closed pore as a reference structure. Each subunit was compared to the reference structures using a sliding window and the average RMSD was calculated and plotted against residue number. Using this approach the presence of a kink can be detected by an increase in RMSD around the residue where the kink occurs. Note that this approach however does not allow us to identify the position of the kink but only detect its presence. The analysis resulted in 125 RMSD vs residue data sets (5 subunits * 25 frames) for each structure and the presence of a kink was defined as an RMSD value above a given cutoff of 1.5 Å. The % occurrence of kinks in the 3 groups of structures was calculated from the RMSD vs residue data sets.

Implementation of restraints

Distance restraints

Table 2 and Figure 2 in the main article summarise the raw experimental data used for the restraints and depicts the position of all residues for both accessibility and distance restraints. For clarity, the residues are only shown on 1 of the 5 subunits but FRET and EPR experiments are carried out with fully labelled proteins where each subunit contains a fluorescent or magnetic tag. The inter-subunit distance hence represents the distance between equivalent residues in neighbouring subunits or diagonally related subunits. Based on the pentameric structure a single distance measurement gives rise to a total of 10 distance restraints, 5 from neighbouring subunits and 5 from diagonal subunits.

FRET is more accurate for measuring changes in distances rather than absolute distances. Consequently, data from FRET experiments was reported as the difference between the

inter-subunit distance in the closed state (d_{closed}) and the same distance in the open state (d_{open}) given by

$$d_{open} = d_{closed} + \Delta d_{FRET} \tag{1}$$

where Δd_{FRET} is the change in distance calculated from the FRET experiment. The restraint was implemented using a flat bottom harmonic potential given by:

$$V(r_{ij}) = \begin{cases} \frac{1}{2}k_{max}(r_{ij} - r_0)^2 & \text{for } r_{ij} < r_0 \\ 0 & \text{for } r_0 \leq r_{ij} \leq r_1 \\ \frac{1}{2}k_{max}(r_{ij} - r_0)^2 & \text{for } r_1 < r_{ij} \end{cases} \tag{2}$$

where r_{ij} is the distance between particle i and particle j at any given time in the simulation, k_{max} is the force constant and r_0 and r_1 are defined by the uncertainty of the inter-subunit distance in the FRET experiment such that $r_0 < d_{open} < r_1$. Considering the geometry of the pentameric structure of MscL each of the 9 reported inter-subunit distance resulted in 10 target distances giving a total of 90 distance restraints from FRET experiments. The target distances used for the simulations are listed in Table 1 in [4].

In EPR experiments inter-subunit distances are reported by the proximity parameter Ω, which is only suitable for semi-quantitative analysis. Distances are categorised as far, intermediate and close based on their Ω values where $\Omega = 1$ means that the distance between the residues is > 15 Å. The data reported [5] shows that all 65 residues in TM1 and TM2 have values of $\Omega = 1$ in the open state making all inter-subunit distances in the open state > 15 Å. The restraints were implemented using harmonic half potentials given by:

$$V(r_{ij}) = \begin{cases} \frac{1}{2}k_{max}(r_{ij} - r_0)^2 & \text{for } r_{ij} < 15\text{Å} \\ 0 & \text{otherwise} \end{cases} \tag{3}$$

As for the distance restraints from FRET data each inter-subunit distance results in 10 restraints giving a total of 650 distance restraints for residues in TM1 and TM2. All distance restraints were incorporated into the GROMACS topology of the CG model of the closed channel using tabulated potentials.

Solvent restraints

Solvent restraints were based on the lipid and water accessibility measurements of residues in the TM helices and the periplasmic loop from EPR experiments. The raw experimental data was converted into restraints by changing the interactions of the side chain particles with water to represent the change in solvent exposure of selected residues upon opening of the channel.

In EPR experiments lipid exposure of specific residues is measured by the collision frequency of the probe with lipid accessible O_2 and is reported by the parameter $\Delta P_{1/2}O_2$, representing

the change in lipid exposure upon channel opening. Similarly, water exposure of residues is measured by monitoring the collision with water soluble NiEDDA and is represented by the parameter $\Delta P_{1/2}NiEDDA$. Although lipid and water accessibility are reported separately they are conceptually related and they can be considered as two opposing trends of the same phenomena i.e. an increase in water exposure can be seen as a decrease in lipid exposure. To simplify the conversion of the lipid and water accessibility data into restraints we combined the data from the two measurements into a single parameter called $\Delta hydrophobic$. This parameter represents a change in hydrophobic character of a given residue upon opening of the channel. Hence an increase in lipid exposure, reported as a positive $\Delta P_{1/2}O_2$ to reflect the increased collision frequency, is equivalent to a increase in hydrophobic character. A decrease in lipid exposure represents a decrease in hydrophobicity. In contrast, an increase in water exposure, reported as a positive $\Delta P_{1/2}NiEDDA$ reflects a decrease in hydrophobic character and a decrease in water accessibility means an increase in hydrophobic character. Since we are interested in the change in hydrophobic character upon opening of the channel the following relationship holds:

$$\Delta\Delta P_{1/2}O_2 = \Delta hydrophobic = -\Delta\Delta P_{1/2}NiEDDA \tag{4}$$

where $\Delta\Delta P_{1/2}O_2$ and $\Delta\Delta P_{1/2}NiEDDA$ are the difference in $\Delta P_{1/2}O_2$ and $\Delta P_{1/2}NiEDDA$ between the closed and the open pore structure as reported from the EPR experiments. A positive value of $\Delta hydrophobic$ corresponds to an increase in hydrophobic character while a negative $\Delta hydrophobic$ means the residue experiences a decrease in hydrophobic character. Using the reported values for $\Delta P_{1/2}NiEDDA$ and $\Delta P_{1/2}O_2$, $\Delta hydrophobic$ was determined for 54 residues in TM1 and TM2 [5] and 28 residues in the periplasmic loop [3] (see Table 2 in the main article). Some residues showed no or little change in hydrophobic character and, as a guideline, residues with $|\Delta hydrophobic| < 3.0$ were not used for restraints. A total of 43 residues in TM1 and TM2 and 10 residues in the periplasmic loop were selected resulting in a total of 265 solvent restraints.

The next step was to incorporate $\Delta hydrophobic$ into the MARTINI force field. In the MARTINI force field the strength of non-bonded interactions are modelled using a Lennard-Jones potential. The strengths of the attraction or repulsion between particles is described by interaction levels ranging from supra attractive (0) to super repulsive (IX) (see Table 1 in [6]). To implement the solvent restraints the value of $\Delta hydrophobic$ for the selected residues was used to increase or decrease the interaction level of the side chain particles with water particles (type P4). Based on the spread of $\Delta hydrophobic$ values among the 43 residues, we divided the residues into 3 groups: i) residues with a $\Delta hydrophobic$ between 3 and 6 were assigned a change in interaction level of 1, ii) for residues with $\Delta hydrophobic$ between 7 and 18 a change of interaction level of II and iii) a change in interaction level of III was used for residues with $\Delta hydrophobic$ larger than 18. This assignment is a compromise between introducing a change in the interaction to model the restraint without introducing a change that is too large for a given residue type. According to the above definition of $\Delta hydrophobic$ and the interaction matrix of the MARTINI force field a negative $\Delta hydrophobic$ value (a

decrease in hydrophobic character) will result in a decrease of interaction level between water and the selected residue. Using these changes in interaction level as a guideline, the $\Delta hydrophobic$ for each residue was mapped to a change in interaction level to create a new set of particle types which contained the altered interaction levels with water but unaltered interactions with all other particle types. The residues Gly22, Leu19 are used as examples to illustrate the changes in interaction level. In the MARTINI force field water is modelled as a particle type P4 while in the MscL protein, based on residue type and secondary structure, Gly22 is modelled as particle type. N0 with an interaction level IV between water (P4) and Gly22 (N0) as defined in the Interaction matrix of the MARTINI force field (see Table 1 in [6]). Gly22 shows a $\Delta hydrophobic$ value of 7 and hence its interaction level with water was increased from IV to VI. Similarly, the interaction level of Leu19 which is modelled as a particle type C1 was decreased from VIII to V, based on its $\Delta hydrophobic$ value of -21. Tables S1 to S3 show the changes in interaction levels and the new particle types for the solvent restraints in TM1, TM2 and the periplasmic loop.

Introducing restraints during simulation

The final simulation system contained a total of 740 distance restraints and 265 solvent restraints that describe the changes in inter-subunit distances and solvent accessibly between the closed and the open pore structure. Hence, the restraints represent a gradual change in the MscL structure during the gating of the channel. We therefore aimed to slowly introduce the restraints during the simulation rather than impose a sudden change onto the closed structure. This was achieved using the free energy perturbation (FEP) method in GROMACS. A coupling parameter λ is used to linearly interpolate between two states of the simulation system. During the simulation λ is slowly increased from $\lambda = 0$, where no restraints are present to $\lambda = 1$ where restraints are at their maximum. In the case of the distance restraints this means that the force constant of the harmonic potentials is slowly increased from k = 0 at $\lambda = 0$ to k_{max} at $\lambda = 1$. Similarly, for the solvent restraints the side chain particles of the restrained residues is slowly morphed from the normal particle type ($\lambda = 0$) into the new particle type ($\lambda = 1$). This means the interaction level of the side chain particle with water is slowly increased or decreased.

References

1. Sukharev S, Durell S, Guy H (2001) Structural models of the mscl gating mechanism. Biophys J 81: 917-936.

2. Sukharev S, Betanzos M, Chiang C, Guy H (2001) The gating mechanism of the large mechanosensitive channel mscl. Nature 409: 720-724.

3. Meyer G (2007) Electro Paramagnetic Resonance Spectroscopy and Computer Modelling Studies of the Structure of Prokaryotic Mechanosensitive Channels. Ph.D. thesis, The University of Queensland, Brisbane, Australia.

4. Corry B, Hurst A, Pal P, Nomura T, Rigby P, et al. (2010) An improved open-channel structure of mscl determined from fret confocal microscopy and simulation. J Gen Physiol 136: 483-494.

5. Perozo E, Cortes D, Sompornpisut P, Kloda A, Martinac B (2002) Open channel structure of mscl and the gating mechanism of mechanosensitive channels. Nature 418: 942-948.

6. Marrink S, Risselada H, Yefimov S, Tieleman D, de Vries A (2007) The MARTINI force field: Coarse grained model for biomolecular simulations. J Phys Chem B 111: 7812-7824.

Table S1: Changes to the interactions between selected side chain particles and water particles to implement solvent restraints for TM1. Δhydrophobic is based on lipid and water accessibility from EPR [5]. Normal Particle type and Interaction levels refers to the standard particle types in the Martini force field and its interaction with water particles (type P4) [6]. New Particle type and Interaction level refers to the newly created particle types that have altered interactions with water particles based on Δhydrophobic (P4) but unaltered interactions with all other particle types.

Residue No	Residue type	Δhydrophobic	Normal Particle Type	Normal Interaction level with P4	New Particle Type	New Interaction level with P4
14	GLY	3.5	P5	0	P51	I
15	ASN	-1.0	P5	0	-	-
16	VAL	-5.0	C2	VII	A26	VI
17	VAL	0.0	C2	VII	-	-
18	ASP	3.0	Qa	0	Qa1	I
19	LEU	-21.0	C1	VIII	A15	V
20	ALA	-3.5	C5	V	C54	IV
21	VAL	-7.0	C2	VII	A26	VI
22	GLY	7.0	N0	IV	N06	VI
23	VAL	-7.0	C2	VII	A25	VI
24	ILE	-7.0	C1	VIII	A16	VI
25	ILE	-21.0	C1	VIII	A15	V
26	GLY	-5.0	N0	IV	N03	III
27	ALA	-6.5	C5	V	C54	IV
28	ALA	4.0	C5	V	C56	VI
29	PHE	-3.3	SC4	VI	S45	V
29	PHE	-3.3	SC4	VI	S45	V
29	PHE	-3.3	SC4	VI	S45	V
30	GLY	-6.0	N0	IV	N03	III
31	LYS	4.0	Qd	0	Qd1	I
32	ILE	-8.0	C1	VIII	A16	VI
33	VAL	-5.0	C2	VII	A26	VI
34	SER	-4.0	P1	II	P11	I
35	SER	-3.5	P1	II	P11	I
36	LEU	-4.0	C1	VIII	A17	VII
37	VAL	-3.5	C2	VII	A16	VI
38	ALA	-16.0	N0	IV	N02	II
39	ASP	0.0	Qa	0	-	-
40	ILE	-5.0	C1	VIII	A17	VII
41	ILE	6.5	C1	VIII	-	- [a]
42	MET	-18.0	C5	V	C52	II

[a] level VIII is already the highest interaction level of any particles with water (type P4). higher levels are only used for charged particles

Table S2: Changes to the interactions between selected side chain particles and water particles to implement solvent restraints for TM2. Δhydrophobic is based on lipid and water accessibility from EPR [5]. Normal Particle type and Interaction levels refers to the standard particle types in the Martini force field and its interaction with water particles (type P4) [6]. New Particle type and Interaction level refers to the newly created particle types that have altered interactions with water particles based on Δhydrophobic (P4) but unaltered interactions with all other particle types.

Residue No	Residue type	Δhydrophobic	Normal Particle Type	Normal Interaction level with P4	New Particle Type	New Interaction level with P4
72	VAL	5	C2	VII	A28	VIII
73	MET	-29	C5	V	C52	II
74	HIS	-1.5	SC4	VI	S45	V
74	HIS	-1.5	SP1	II	Z12	II
74	HIS	-1.5	SP1	II	Z12	II
75	TYR	-4	SC4	VI	S45	V
75	TYR	-4	SC4	VI	S45	V
75	TYR	-4	SP1	II	Z11	I
76	GLY	1.5	N0	IV	-	-
77	VAL	-17.5	C2	VII	A25	V
78	PHE	-2.7	SC4	VI	S45	V
78	PHE	-2.7	SC4	VI	S45	V
78	PHE	-2.7	SC4	VI	S45	V
79	ILE	-2	C1	VIII	-	-
80	GLN	-2	P4	0	-	-
81	ASN	2	P5	0	-	-
82	VAL	-2	C2	VII	-	-
83	PHE	1	SC4	VI	-	-
84	ASP	3	Qa	0	Qa1	I
85	PHE	1.8	SC4	VI	S47	VII
85	PHE	1.8	SC4	VI	S47	VII
85	PHE	1.8	SC4	VI	S47	VII
86	LEU	4.5	C1	VIII	A18	VIII
87	ILE	3	C1	VIII	A18	VIII
88	VAL	7	C2	VII	A28	VIII
89	ALA	8.5	C5	V	C57	VII
90	PHE	2	SC4	VI	-	-
91	ALA	-3	C5	V	C54	IV
92	ILE	8	C1	VIII	A18	VIII
93	PHE	2	SC4	VI	-	-
94	MET	5.5	C5	V	C56	VI
95	ALA	6.5	N0	IV	N06	VI
96	ILE	5.5	C1	VIII	A18	VIII

Table S3: Changes to the interactions between selected side chain particles and water particles to implement solvent restraints for the periplasmic loop. Δhydrophobic is based on lipid and water accessibility from EPR [5]. Normal Particle type and Interaction levels refers to the standard particle types in the Martini force field and its interaction with water particles (type P4) [3]. New Particle type and Interaction level refers to the newly created particle types that have altered interactions with water particles based on Δhydrophobic (P4) but unaltered interactions with all other particle types.

Residue No	Residue type	Δhydrophobic	Normal Particle Type	Normal Interaction level with P4	New Particle Type	New Interaction level with P4
44	PRO	-3.0	C2	VII	A26	VI
45	LEU	1.5	C1	VIII	-	-
46	GLY	0.2	P5	0	-	-
47	LEU	-0.8	C1	VIII	-	-
48	LEU	-0.6	C1	VIII	-	-
49	ILE	-2.1	C1	VIII	-	-
50	GLY	-2.2	P5	0	-	-
51	GLY	3.4	P5	0	P51	I
52	ILE	1.2	C1	VIII	-	-
53	ASP	1.7	Qa	0	-	-
54	PHE	-0.6	SC4	VI	-	-
55	LYS	1.5	C3	VI	C37	VII
55	LYS	1.5	Qd	0	C37	VII
56	GLN	2.3	P4	0	C37	VII
57	PHE	-1.2	SC4	VI	-	-
58	ALA	10.1	P4	I	P43	III
59	VAL	-2.5	C4	VI	-	-
60	THR	3.2	P1	II	P13	III
61	LEU	0.6	C1	VIII	-	- [a]
62	ARG	1.3	Qd	0	-	-
62	ARG	1.0	N0	IV	-	-
63	ASP	3.8	Qa	0	Qa1	I
64	ALA	5.5	P4	0	P42	II
65	GLN	0.9	P4	0	-	-
66	GLY	3.8	P5	0	P51	I
67	ASP	1.4	Qa	0	-	-
68	ILE	5.4	C1	VIII	-	- [a]
69	PRO	1.3	C2	VII	-	-
70	ALA	0.7	P4	0	-	-
71	VAL	-0.2	C2	VII	-	-

[a] level VIII is already the highest interaction level of any particles with water (type P4). higher levels are only used for charged particles
